# Analytical Methods for Quantification and Identification of Intact Glucosinolates in *Arabidopsis* Roots Using LC-QqQ(LIT)-MS/MS

**DOI:** 10.3390/metabo11010047

**Published:** 2021-01-11

**Authors:** Kourosh Hooshmand, Inge S. Fomsgaard

**Affiliations:** Department of Agroecology, Aarhus University, DK-4200 Slagelse, Denmark; Kourosh.Hooshmand@agro.au.dk

**Keywords:** intact glucosinolates, reversed-phase liquid chromatography (RPLC), triple quadrupole-linear ion trap mass spectrometry, multiple reaction monitoring (MRM), method validation, glucosinolate profiling

## Abstract

Glucosinolates are biologically active secondary metabolites in Brassicaceae plants that play a critical role in positive and negative interactions between plants and root-associated microbial communities. The aim of this study was to develop a reversed-phase liquid chromatography method to quantify and identify intact glucosinolates in the root of *Arabidopsis thaliana* (*Arabidopsis*) grown in non-sterile natural soil by using liquid chromatography-hybrid triple quadruple-linear ion trap (LC-QqQ(LIT)) mass spectrometry. The Synergi Fusion C18-based column was found to be effective for sufficient retention and separation of nine intact glucosinolates without the need for time-consuming desulfation or ion-pairing steps. Method validation results showed satisfactory inter-day and intra-day precision for all glucosinolates except for 4-hydroxyglucobrassicin. Good inter-day and intra-day accuracy and recovery results were observed for glucoiberin, gluconapin, glucobrassicin, 4-methoxyglucobrassicin and neoglucobrassicin. However, for 4-hydroxyglucobrassicin, glucoraphanin and glucoerucin corrections to quantification results might be necessary since the recovery and accuracy results were not optimal. Matrix effects were shown to have a negligible effect on the ionization of all target analytes. The established liquid chromatography–tandem mass spectrometry (LC-MS/MS) method was applied to quantify target intact glucosinolates in *Arabidopsis* root crude extract of four different wild-type accessions where differences in terms of concentration and composition of intact glucosinolates were observed. Employment of sensitive and selective precursor ion survey scan of *m*/*z* 97 in combination with the information-dependent acquisition (IDA) of the enhanced product ion (EPI) dependent scan (Prec97-IDA-EPI) using LC-QqQ(LIT) provided high confidence in structural characterization of diverse intact glucosinolate profiles in complex *Arabidopsis* root crude extract.

## 1. Introduction

**Figure 1:**
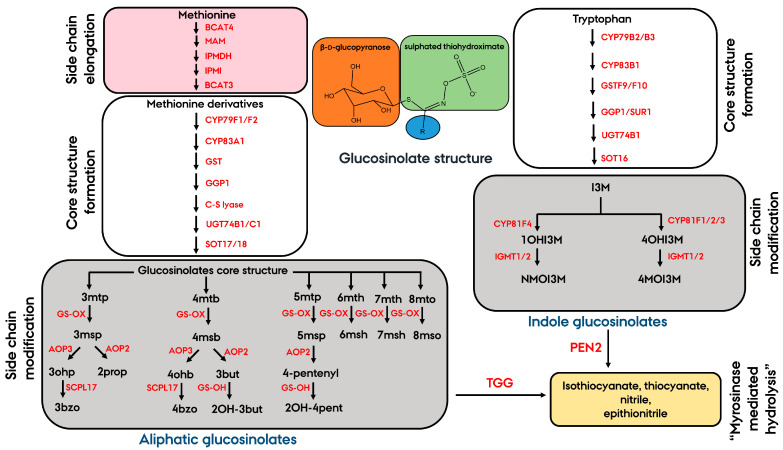
Schematic representation of the biosynthetic pathway leading to aliphatic glucosinolates, indole glucosinolates and their respective hydrolysis products in *Arabidopsis* thaliana root. Arrows between compounds represent the major enzymes (in red font) involved in the biosynthesis of glucosinolates. Glucosinolates are characterized by having a common core structure containing a β-d-glucopyranose residue (orange background) linked to a sulfated thiohydroximate (green background) with a variable side chain R group (blue background). Side-chain elongation of the precursor amino acid methionine (one to six methylene groups are sequentially inserted into the methionine side chain) is shown on a dark pink background. Glucosinolate side-chain modification is shown on a grey background. Glucosinolates are released from the vacuole and brought into contact with endogenous thioglucosidases called myrosinases such as Penetration2 (PEN2) or β-thioglucoside glucohydrolases (TGG) that mediate the degradation of glucosinolates to a variety of products, including isothiocyanates, thiocyanates, nitriles, and epithionitriles (yellow background). Abbreviations for intact glucosinolates and full names: 3mtp, 3-methylthiopropyl glucosinolate; 3msp, glucoiberin; 3ohp, 3-hydroxypropyl glucosinolate; 2prop, sinigrin; 3bzo, glucomalcomiin; 4mtb, glucoerucin; 4msb, glucoraphanin; 4ohb, 4-hydroxybutyl glucosinolate; 4bzo, 4-benzoyloxybutyl; 3but, gluconapin; 2OH-3but, progoitrin; 5mtp, 5-methylthiopentyl glucosinolate; 5msp, 5-methylsulfinylpentyl glucosinolate; 4-pentenyl, 4-pentenyl glucosinolate; 2OH-4pent, 2-hydroxy-4-pentenyl (or gluconapoleiferin); 6mth, 6-methylthiohexyl (or glucolesquerellin); 6msh, 6-methylsulfinylhexyl (or glucohesperin); 7mth, 7-methylthioheptyl glucosinolate; 7msh, 7-methylsulfinylheptyl (or glucoibarin); 8-mto, 8-methylthiooctyl glucosinolate; 8mso, 8-methylsulfinyloctyl (or glucohirsutin); I3M, glucobrassicin; 1OHI3M, 1-hydroxyglucobrassicin; 4OHI3M, 4-hydroxyglucobrassicin; NMOI3M, neoglucobrassicin; 4MOI3M, 4-methoxyglucobrassicin.

Glucosinolates are anionic sulfur- and nitrogen-containing plant secondary metabolites that are largely limited to species within the family Crucifereae, including the model plant *Arabidopsis thaliana* (hereafter *Arabidopsis*) [1]. Glucosinolates are characterized by having a common core structure containing a β-d-glucopyranose residue linked to a sulfated thiohydroximate (Figure 1). Depending on the amino acid precursor of the variable side chain (R group), glucosinolates can be grouped into three chemical classes: (I) aliphatic glucosinolates derived from methionine, (II) indole glucosinolates derived from tryptophan and (III) aromatic glucosinolates derived from phenylalanine or tyrosine [2]. The biosynthesis of glucosinolates occurs in three independent steps; side-chain elongation (for methionine and phenylalanine-derived glucosinolates), core structure formation and secondary modification (Figure 1) [3]. Aliphatic glucosinolates undergo a wide range of secondary transformations, including oxidation, hydroxylation, alkenylations and benzoylations, while for indole glucosinolates, secondary transformations include only hydroxylations and methoxylations (gray background in Figure 1) [3]. Extensive amino acid elongation and glucosinolate side-chain modification contribute to a large structural diversity of the glucosinolate profile. Diversification of glucosinolate profile in plants has been shaped by natural selection as a consequence of diverse ecological and physiological processes during the course of evolution [4,5].

Reversed-phase liquid chromatography (RPLC) represents the most widely used and preferred separation technique for the analysis of glucosinolates of all structural classes [6]. Glucosinolates are generally subjected to time-consuming sulfatase treatment prior to RPLC analysis [7]. This leads to cleavage of the sulfate moiety that converts intact glucosinolates into desulfo-glucosinolates, thereby the separation of glucosinolates by reversed-phase chromatography is facilitated due to their reduced polarity compared to the intact form. However, sulfatase treatment requires a laborious sample-processing step using anion-exchange chromatography even though it results in cleaner sample extracts. Thus, separation of glucosinolates in their intact form on RPLC columns with minimal sample preparation remains the preferred and efficient approach even though in several cases it posed a challenge due to the presence of the sulfated group, which made the chromatography separation difficult [7,8,9].

Only a limited number of glucosinolate reference compounds are commercially available. Detection and quantification of glucosinolates by low-resolution mass spectrometry can therefore be challenging. A low-resolution mass spectrometry method that can reliably characterize structurally diverse glucosinolates in the sample is needed. Application of Information Dependent Acquisition (IDA) methods with hybrid triple quadrupole-linear ion trap QqQ(LIT) instruments made structural elucidation of metabolites with similar structure possible, both in drug discovery [10] and in the analysis of triterpene saponins [11]. Prec scans were employed by Qiao et al. [12] for detecting metabolites with diverse structures that produced similar product ions as a result of sharing identical structural moieties. Intact glucosinolates with variable side chains under normal collision energy conditions consistently generate the *m*/*z* ions 96 and 97 corresponding to [SO_4_^−^] and [HSO_4_^−^], respectively, as major product ions. However, the characteristic ion *m*/*z* 97 could not discriminate glucosinolates completely from other sulfate-containing metabolites such as flavonols or sulfated sterols [7,13]. Hence, additional confidence for tentative identification of glucosinolates can be achieved by considering a highly specific characteristic *m*/*z* 259 ion corresponding to sulfated glucose and a glucose moiety characteristic product ion at *m*/*z* 275, both as the minor fragments (Supplementary Figure S1) which are formed from intact glucosinolates through intramolecular rearrangements [13].

The purpose of this study was to develop and validate an RPLC-tandem mass spectrometry method for the chromatographic separation and simultaneous qualitative and quantitative analysis of major intact glucosinolates (with a variable side chain) in roots of plants growing in natural soil. Liquid chromatography-tandem mass spectrometry (LC-MS/MS) method development was performed on nine intact glucosinolates (including internal standard), while quantification and validation were performed for eight glucosinolates (excluding internal standard). The method was applied to the methanolic root extract of four different *Arabidopsis* accessions to determine the naturally occurring genetic variation in glucosinolate profiles and concentrations. Furthermore, the purpose of this study was to make a novel analytical method using Precursor ion information-dependent acquisition (IDA) of the enhanced product ion (Prec-IDA-EPI) on a QqQ(LIT) instrument for tentative identification and profiling of the naturally occurring glucosinolates of all structural classes in the root extract of *Arabidopsis*.

## 2. Results and Discussion

### 2.1. LC-MS/MS Method Development for Separation of Intact Glucosinolates

**Figure 2:**
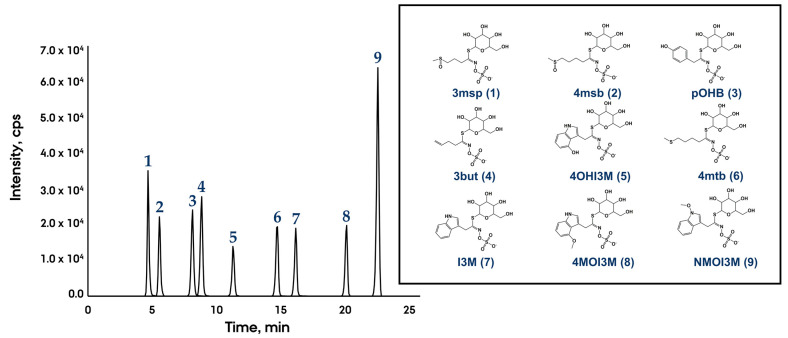
Chromatogram displaying the separation of intact glucosinolates with a variable side chain (100 ng/mL standard mixture) by liquid chromatography and detection by tandem mass spectrometry in multiple reaction monitoring (MRM) mode on a Synergi 4 µm Fusion-RP (250 × 2 mm) using MeOH/water with 0.1% acetic acid as solvent system. Sinalbin (pOHB) was used as an internal standard. The numbers on top of the chromatogram correspond to their associated structures, which are displayed on the right side of the chromatogram. Full names and abbreviations: glucoiberin (3msp), glucoraphanin (4msb), sinalbin (pOHB), gluconapin (3but), 4-hydroxyglucobrassicin (4OHI3M), glucoerucin (4mtb), glucobrassicin (I3M), 4-methoxyglucobrassicin (4MOI3M) and neoglucobrassicin (NMOI3M). 3msp, 3but, 4msb and 4mtb are aliphatic glucosinolates, while I3M, 4OHI3M, 4MOI3M and NMOI3M are indole glucosinolates.

Three different columns with various C18 reversed-phase stationary phases in combination with four different solvent systems (MeOH/water/acetic acid; MeOH/water/formic acid; ACN/water/acetic acid; ACN/water/formic acid) (method Section 3.4) were tested in order to optimize the separation of nine intact glucosinolates (including internal standard) (Figure 2). The first chromatographic separation was performed using a Kinetex 2.6 µm XB-C18 (100 × 2.1 mm) (Kinetex XB) column with ACN/water with 0.05% formic acid, as explained in Crocoll et al. [7] except that the particle size of the column we used was 2.1 µm instead of 1.7 µm. Even though a good separation was observed for most of the target glucosinolates, 3msp (1) (retention time 0.64 min) and 4msb (2) (retention time 0.71 min) were not well resolved (Supplementary Figure S2). In addition, the peak intensity for several glucosinolates was low (Supplementary Figure S2). Besides, peak tailing for some of the glucosinolates (4msb (2), pOHB (3), 3but (4) and 4OHI3M (5)) was observed (Supplementary Figure S2), probably due to additional unwanted secondary interactions of the analytes with the free silanol groups of the column. Furthermore, early eluting peaks, 3msp (1) and 4msb (2) eluted from the column shortly after the dead time (0.62 min, estimated by calculation). Due to the presence of numerous co-eluting small molecules, this region is susceptible to severe matrix effect leading to sensitivity suppression and poor reproducibility. Hence, this chromatographic method was not selected.

The second chromatographic system was based on a Synergi 4 µm Fusion-RP (250 × 2 mm) (Fusion) analytical column with a C18 polar embedded functionality. All four solvent systems were tested. The best results in terms of separation and peak symmetry were obtained by using segmented gradients with water as mobile phase A and methanol as B at 40 °C. Improved separation, narrow peak shapes with minimal tailing and optimal ionization (maximum sensitivity) were achieved by using acetic acid (0.1%) as a modifier (pH = 3.3) (Figure 2 and Supplementary Figure S3A). In contrast, the addition of 0.1% formic acid in both mobile phases A and B (pH = 2.7) led to strong ion signal suppression in negative electrospray ionization (ESI) mode for the majority of the target compounds (around 6 times lower peak intensity was observed for several glucosinolates than those observed with acetic acid) (Supplementary Figure S3B). Hence, the ionization process of glucosinolates in negative ESI mode seemed to be pH-dependent. Our results are consistent with the results of two different studies showing that the addition of acetic acid in mobile phases was more effective than formic acid in enhancing the signal-to-noise ratio of phenolic acids and androgen receptor modulators in negative ion mode [14,15]. The underlying cause of these effects was correlated with the lower gas-phase proton affinity of formic acid compared to acetic acid in the negative ion mode, resulting in a dramatic supersession of the analytes signal due to lack of negative charges on the surface of the ESI droplet [15].

The dead time (retention time of a non-retained compound) of the Fusion RP column was determined by using uracil according to Snyder et al. [16], which resulted in t_0_ = 1.9 min. Thus, highly polar glucosinolates like 3msp (1) (XLogP3-AA = −2.4) could be retained sufficiently in the Fusion column (RT = 4.8 min) (Figure 2). Our results contradicted the observation of Ares and co-workers [9], where an unsatisfactory separation was observed in a Fusion column, while our method provided adequate analyte retention and peaks were well resolved. Ares et al., 2013 study did not report the chromatographic conditions that led to undesirable results [9]; hence, we are not able to explain the reasons for the differences in the results of Ares et al., 2013 and our results.

The chromatographic method using the methanol and acetic acid-based mobile phase (Figure 2) in the Fusion column showed the ability to adequately separate the most polar and structurally similar glucosinolates such as 3msp (1) and 4msb (2) (differ only in one side-chain methylene group) [8]. The use of water and acetonitrile as elution solvents together with acetic acid (0.1%), resulted in satisfactory resolution and sensitivity (Supplementary Figure S3C). However, 3msp (1) and 4msb (2) peaks were not completely separated at the baseline, likely due to the higher elution strength of acetonitrile than methanol, which did not allow proper retention of those analytes in the column. However, the optimal separation of isobaric glucosinolates like NMOI3M and 4MOI3M (for which the optimal quantification multiple reaction monitoring (MRM) transition was the same) were achieved with both methanol and acetonitrile-based mobile phases (Supplementary Figure S3A,C).

Kinetex XB and Synergi 4 µm Polar-RP (250 × 2 mm) (Polar) columns are used in many publications for chromatographic separation of polar natural compounds. Therefore, the solvent system used above with excellent results in the Fusion column (MeOH/water/acetic acid) was subjected to a final test in the Polar and the Kinetex XB. No changes in terms of elution order of glucosinolates occurred in all three columns tested (Supplementary Figure S4). However, the retention of glucosinolates was much stronger on the Fusion than on the Polar column (both columns have the same length and internal diameter but different stationary phase materials). While the column dead time for both Fusion and polar columns was about 1.9 min, the first eluting peak, 3msp (1), eluted at 4.5 min in Fusion and 2.6 min in polar column (Supplementary Figure S4A,B). Thus 3msp was sufficiently retained in the Fusion column, whereas it eluted the Polar column near the dead time. No peak for 4OHI3M (5) was observed on the Polar column as well as very low peak intensity was observed for 4OHI3M on the Kinetex XB column (Supplementary Figure S4B,C). This suggests that 4OHI3M might have been strongly retained on both columns. Furthermore, the Polar column exhibited unsatisfactory retention and resolution for early eluting peaks (3msp (1), 4msb (2), pOHB (3) and 3but (4)), while the highest efficiency of separation for all peaks was achieved when using Fusion and Kinetex XB columns (Supplementary Figure S4A–C). In summary, our result showed a baseline separation of nine intact glucosinolates (Figure 2) by using a C18 Synergi Fusion column where the peaks were uniformly distributed over the elution window of 5 to 23 min as well as maximum sensitivity for quantitative analysis could be achieved compared to other analytical columns tested. Hence, this Fusion column was used for further analysis.

### 2.2. Method Validation of the Final LC-MS/MS Quantitation Method

Specific guidelines for validation of analytical methods aimed at being used for the analysis of plant secondary metabolites in the agroecological context do not exist. The level of validation (which performance characteristics are included) of such analytical methods reported in the literature vary substantially. The performance characteristics evaluated here were based on the Eurachem guideline “The Fitness for Purpose of Analytical methods” [17] and the European guideline for the validation of methods used for the analysis of pesticide contamination in food (SANTE/11813/2017) [18]. The validation tests were performed by spiking a mixed solution of eight glucosinolates into a blank matrix; six biological replicates at each of three concentration levels (materials and methods Section 3.6). Sinalbin (pOHB) was not included in the validation study due to its role as an internal standard. Extraction efficiency (extraction recovery) was based on the mean peak area of standards spiked before extraction in relation to the mean peak area of standards spiked to the blank matrix after extraction (materials and methods Section 3.6). Extraction recovery for three concentration levels was found to be between 36–145%, respectively (Table 1). The extraction recovery results obtained for 3msp, 3but, I3M, 4MOI3M and NMOI3M at three spiked concentration levels ranged from 78% to 98%, which falls inside the recommended range of 70–120% for analysis of pesticide residues in food (SANTE/11813/2017) [18]. Thus, the sample treatment applied for quantitative extraction of those compounds was appropriate. The recovery for 4msb, 4mtb and 4OHI3M was outside the mentioned range. 4OHI3M yielded low recoveries of 41–46% at all three spiked concentration levels. For 4mtb, the recovery varied across concentration levels (ranged between 36 to 65%), while for 4msb recovery values were above 120% (Table 1). The low recoveries for 4OHI3M might stem from the thermal degradation of this thermo-labile compound during sample treatment where the samples were subjected to the heating at 70 °C to prevent a potential myrosinase-mediated glucosinolate breakdown, consistent with previous reports [19,20,21]. In addition, among all the indole glucosinolates tested (I3M, 4MOI3M, NMOI3M and 4OHI3M) (Figure 2), only 4OHI3M yielded low recovery values. The presence of a hydroxyl functional group influences the thermal stability of indole glucosinolate when they are subjected to heat treatment [21]. The fate of 4OHI3M after heat treatment was not previously shown in the literature and more studies are needed to identify 4OHI3M degradation products. Within the aliphatic glucosinolates, 4mtb (reduced form of 4msb, Figure 1 and Figure 2) was susceptible to degradation at the lowest and medium spiking level probably due to an oxidation reaction, which turned 4mtb into its oxidized form, 4msb. Hence, the high recovery observed for 4msb is correlated with the heat-induced oxidation of 4mtb to 4msb. The notion that heat treatment promotes the oxidation of 4mtb is in agreement with the observation made by Hanschen et al. [21]. Our results contradicted the results observed by Ares et al. [22] where satisfactory recovery values were obtained for 4mtb and 4msb after diluting the samples with heated water (70 °C) during the sample treatment [22]. Hence, reduced extraction recovery for 4mtb and 4msb in our result might be correlated with a longer heat treatment period (10 min) [22] and reduction of heat treatment process time may improve the recovery values in future studies [21,22]. Despite low and high recovery observed for 4mtb, 4msb and 4OHI3M, the RSD of the method remained ≤20% using six replicate extract (Table 1). According to the pesticide contamination guideline SANTE/11813/2017, a recovery higher than 30% or below 140% can be accepted if the values are consistent and reproducible (RSD ≤ 20%) [18]. However, other guidelines do not specify such limits. We consider recovery values lower than 30% and higher than 140% (as in one case for 4msb) to be acceptable because the results are consistent and reproducible. According to the IUPAC guideline [23], a recovery percentage can be used for correction of the raw data in cases when true values are needed for instance for comparing the analytical results with results from other papers. In our case, the analytical results will be used for comparing concentration levels between *Arabidopsis* accessions in our study and recovery based corrections have not been done.

The influence of the matrix components on the ionization process was assessed according to the description given in SANTE/11813/2017 [18]. Matrix interference was based on tests, in which mean peak areas of standards spiked into blank matrix were compared with mean peak of the standards in solvent (materials and methods Section 3.6). Matrix interference values of 100% were considered as no effect. If the response of the analyte in the matrix (matrix-matched standard) was suppressed or enhanced by more than 20% relative to the solvent-based standard, it was considered to be affected by the matrix effect [18]. There was a weak enhancement of the signal for all the glucosinolates at the medium and highest spiking level (ranging from 105 to 123%), while at the lowest spiking level, the signal was moderately enhanced, as shown in Table 1. Thus, compensation of matrix effect was considered to be necessary for the low concentration level [18]. Even though the matrix effect results met the SANTE/11813/2017 criteria at medium and highest spiking level, we carried out all the quantification using a matrix-matched calibration curve throughout this study.

Tests for determining precision and accuracy were performed by spiking standards into a blank matrix (materials and methods Section 3.6). Inter- and intra-day precision (repeatability) for all the target glucosinolates did not exceed 15% RSD at three concentration levels, except for 4OHI3M where the precision was in the ranges 22–32% and 16–21%, respectively (Table 2). The intra- and inter-day accuracy (relative error (%RE)), was calculated on basis of results obtained in the spiked samples in relation to known spiked concentrations and thus expresses the recovery of the full method. Both intra- and inter-day accuracy results were in line with the results of the extraction efficiency. For 3msp, 3but, I3M, 4MOI3M and NMOI3M the accuracy was inside the range recommended in the SANTE guideline (%RE below 20%), whereas for 4msb, 4mtb and 4OHI3M the accuracies were outside this range (%RE greater than 20%) (Table 2). The close relationship between results for extraction efficiency (extraction recovery) and accuracy (full method recovery) showed that no other factor than the extraction efficiency influenced the accuracy. Matrix effect is commonly another factor that influences the accuracy. However, matrix effects were compensated in this study, because all standard curves were matrix-matched. The importance of using an appropriate internal standard for adequately correcting for the loss of analyte during the sample treatment is clear. The variation in accuracy results may be improved in future studies by the use of isotopically labeled analogs as internal standards, which could correct for the variability in the analyte recovery during sample treatment because the chemical and physical properties of the labeled internal standard are nearly identical to the properties of the unlabeled compound. However, isotopically labeled glucosinolates are not commercially available and the synthesis of properly labeled glucosinolates requires highly specialized skills [24,25,26].

LODs were in the range of 0.04 to 0.19 μg/g and LOQs ranged from 0.13 to 0.62 μg/g (Table 3). The calibration graph for each target analyte was a straight line with coefficient of correlation values (R^2^) above 0.99 in all cases (Supplementary Figure S5). No carry-over effect was observed for any of the analytes of interest, indicating that none of the target intact glucosinolates was retained on the column after separation was complete.

### 2.3. Quantification of Intact Glucosinolates in Arabidopsis Root Extracts by LC-MS/MS

**Figure 3:**
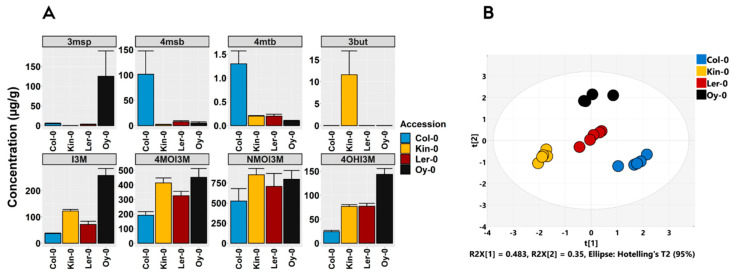
Composition and concentrations of aliphatic and indole glucosinolates in soil-grown *Arabidopsis* accessions (Col-0, Kin-0, Ler-0 and Oy-0) root extracts. (**A**) Concentration (μg/g dry mass). Error bars indicate standard error of the mean of five independent samples (*n* = 5). (**B**) Principal components analysis (PCA) score plot based on glucosinolate concentrations of analyzed *Arabidopsis* root extracts. Different color denotes samples from different *Arabidopsis* accessions (*n* = 5). The ellipse represents the Hotelling T-squared with 95% confidence. Full names and abbreviations: glucoraphanin (4msb), glucoerucin (4mtb), glucoiberin (3msp), gluconapin (3but), glucobrassicin (I3M), 4-methoxyglucobrassicin (4MOI3M), neoglucobrassicin (NMOI3M) and 4-hydroxyglucobrassicin (4OHI3M).

The validated chromatographic method was applied to the methanolic root extract of *Arabidopsis* accessions (Col-0, Kin-0, Ler-0 and Oy-0), known to have genetic variations in their glucosinolate profiles [27]. A total of 8 glucosinolates were identified and quantified (Figure 3A). The PCA plot displayed a clear separation between clusters of *Arabidopsis* accessions along with principal components PC1 and PC2 on the basis of their glucosinolate profile (Figure 3B).

NMOI3M was the main indole glucosinolate in all four accessions accounting for over 45% of the total glucosinolate in each individual accession, whereas the aliphatic glucosinolate (3msp, 3but, 4msb and 4mtb) accumulated in an accession-specific manner. The alkenyl glucosinolate, 3but, was accumulated at a high concentration in Kin-0, while it was not detected in other accessions (Figure 3A). Hence, distinct natural variation in side-chain modifications of aliphatic glucosinolates core structure (conversion of 4msb to 3but) (Figure 1) [3,27] in Kin-0 may serve unique regulatory roles under local adverse environmental conditions [5]. This is in agreement with the observation made by Witzel et al., [28] that the alkenyl glucosinolate, 2-propenyl glucosinolate (2prop) derived from distinct *Arabidopsis* accessions exhibited potent growth inhibitory effect against *Verticillium longisporum* fungal pathogen [28]. In contrast, low alkenyl-accumulating accession, Oy-0 displayed no ability in inhibiting the fungal growth. The exogenous addition of 2prop into the Oy-0 leaf tissue greatly reduced the growth capacity of *Verticillium longisporum* [28], indicating the accession-specific accumulation of alkenyl glucosinolates may play a critical role in suppressing the growth of the soil-borne pathogen.

Oy-0 accumulated a substantially higher concentration of the sulfinylalkyl-derived glucosinolate, 3-msp and the indole glucosinolates, I3M and 4OHI3M in the root compared to other accessions (Figure 3A). A high concentration of 4msb and 4mtb was characteristic of Col-0, whereas Col-0 accumulated a lower concentration of indole glucosinolates compared to other accessions. Ler-0 exhibited a low concentration of aliphatic glucosinolate and a high concentration of indole glucosinolates. The significant differences in the ability of *Arabidopsis* accessions in accumulating distinct glucosinolates suggest that the profile of glucosinolate might have been shaped in those accessions as a consequence of the selection pressures imposed on the plant in their natural habitat which may possess specific regulatory functions [5]. Apart from their direct role in defense against a broad spectrum of soil-borne pathogens [29,30,31,32,33], glucosinolates have been shown to play a critical role in shaping and structuring the root-associated microbiome [34,35]. Hence, further studies are required to uncover the ecological role of glucosinolates in specific *Arabidopsis* accessions [36].

### 2.4. Use of QqQ(LIT) for Simultaneous Tentative Identification of a Range of Intact Glucosinolates in Arabidopsis Roots

The analytical method Prec97-IDA-EPI was optimized first by assuring that all 9 known available glucosinolates were identified. Subsequently, the effectiveness of the method in simultaneous detection and tentative identification of additional compounds from all structural classes of glucosinolates was assessed using a crude extract of *Arabidopsis* root of four different accessions. A total of 20 glucosinolates was putatively identified (Table 4) in all 4 accessions tested. The identity of 8 compounds, for which reference standards were available, was fully verified by comparing the acquired MS/MS spectra against the spectra of the reference compound. Among the 20 compounds, 16 were aliphatic (sulfinylalkyl, thioalkyl, sulfonylalkyl, alkenyl, hydroxyalkenyl and hydroxyalkyl) glucosinolates (Table 4) and 4 were indole glucosinolates. MS/MS fragmentation spectra of all 20 identified and tentatively identified glucosinolates can be found in Supplementary Figure S1. In *Arabidopsis*, about 34 structurally different glucosinolates have been identified to be constitutively present in the seeds and leaves of a collection of accessions, most of which are mainly derived from methionine and tryptophan [27]. Glucosinolate accumulation varies considerably in composition between tissues in *Arabidopsis*. The highest glucosinolate diversity in *Arabidopsis* has been found in siliques, while intermediate diversity are found in leaves and the lowest number of glucosinolates are reported to be found in the roots [37]. *Arabidopsis* produces a huge variety of glucosinolates in root, probably with the purpose of adapting itself to different environmental and ecological conditions and thereby surviving in their very competitive natural habitats [5,36].

**Figure 4:**
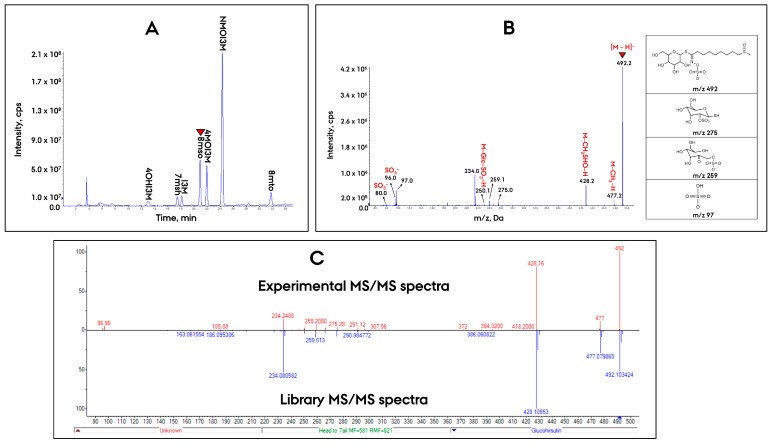
Chromatogram and mass spectra used for the tentative identification of 8-methylsulfinyloctyl glucosinolate (8mso) in Kin-0 root crude extract using the prec97-IDA-EPI method. (**A**) Total ion chromatogram (TIC) of the precursor ion scan was acquired by the data-dependent analysis from Prec97-IDA-EPI. (**B**) EPI scan of the fragmentation profile of 8mso (*m*/*z* 492). (**C**) The head-to-tail comparison of EPI generated MS/MS spectra of [M − H]^−^ at *m*/*z* 492 (red peaks in the top spectrum) from the *Arabidopsis* root extract against the library spectra from the Mass Bank of North America (MoNA) (blue peaks in the bottom spectrum).

The total ion chromatogram (TIC) generated by the Prec97-IDA-EPI scanning method in the crude root extract of Kin-0 accession is displayed in Figure 4A. For the compound, for which its chromatographic peak is shown with a red arrow in Figure 4A, the EPI spectrum is shown in Figure 4B. The spectrum shows the deprotonated molecule *m*/*z* 492 (Figure 4B). A specific diagnostic ion at *m*/*z* 428 corresponded to the neutral loss of the methylsulphinyl group (−64 Da) [M−CH_3_SHO]^−^ [13]. In addition, according to the nitrogen rule, the ion with an even *m*/*z* has an odd number of nitrogen atoms. This provided additional evidence that the [M − H]^−^ deprotonated molecule at *m*/*z* 492 comes from a methionine-derived aliphatic glucosinolate rather than indole glucosinolates (possess two nitrogen atoms in their structure), which further narrow down the number of potential glucosinolate candidates. Annotation of this compound was achieved by submitting the resulting EPI full-scan MS/MS spectra for library searching against a publicly available MS/MS spectral library (the Mass Bank of North America (MoNA)) [38], which assigned the peak as 8-methylsulfinyloctyl glucosinolate (8mso) (Figure 4C). The identification was further validated on basis of chromatographic behavior (retention order) for 8mso (8C carbon side chain length) with XLogP3-AA = −0.1 compared to 3msp (3C) and 4msb (4C) (with XLogP3-AA values of −2.4 and −2.1, respectively), which shows that the more hydrophobic nature of 8mso led to increase in retention on the C18 based Synergi Fusion column (Table 4).

Certain glucosinolate structural isomers such as NMOI3M and 4MOI3M have the same deprotonated molecule *m*/*z* 477 and their fragmentation patterns are very similar. However, the MS/MS spectrum of NMOI3M exhibited a specific diagnostic fragment ion of *m*/*z* 446 derived from the loss of [M−CH_3_O] ion [39], allowing NMOI3M to be readily distinguished from the corresponding 4MOI3M isomer (Supplementary Figure S1). This was further verified by comparing the retention time and MS/MS spectra against the reference standard. Altogether, our results demonstrated that the Prec97-IDA-EPI-based approach using a low-resolution 4500 Q-trap instrument was well suited for tentative identification of a range of intact glucosinolates in a crude extract of *Arabidopsis* root in one chromatographic run while retaining sensitivity, thus making this approach a novel strategy for comprehensive glucosinolates profiling in other Brassica plant species.

## 3. Materials and Methods

### 3.1. Chemicals and Reagents

Chemicals and reagents were obtained from the following commercial sources (purity in parenthesis): Gluconapin (3but) (84%), glucoerucin (4mtb) (90%), glucobrassicin (I3M) (86%), glucoraphanin (4msb) (89%), glucoiberin (3msp) (98%) and internal standard, sinalbin (99%) were purchased from PhytoLab GmbH & Co. KG (Vestenbergsgreuth, Germany). 4-Hydroxyglucobrassicin (4OHI3M) (95%), 4-methoxyglucobrassicin (4MOI3M) (94%) and neoglucobrassicin (NMOI3M) (97%) were acquired from Phytoplan, Diehm & Neuberger GmbH (Heidelberg, Germany). Acetonitrile and methanol (LC/MS grade solvents) were obtained from Fisher Chemical (Roskilde, Denmark). Water was obtained from a MilliQ purifier (Millipore, MA, USA). Glacial acetic acid was purchased from Merck (Darmstadt, Germany).

### 3.2. Plants, Growing Conditions and Harvesting

*Arabidopsis* Col-0, Ler-0, Kin-0 and Oy-0 accessions were chosen for this study. *Arabidopsis* seeds were supplied by the Nottingham *Arabidopsis* Stock Centre (NASC, Loughborough, UK). Col-0 line (NASC stock number N22625) was originally collected from Columbia, Portland, OR, USA, Ler-0 (NASC stock number N97814) was originally collected from Germany, Kin-0 (NASC stock number N22654) was originally collected from Kindalville, MI, USA and Oy-0 (NASC stock number N22658) was originally collected from Oystese, Norway (more information can be found in http://arabidopsis.info/BasicForm by entering each accession NASC stock number). The *Arabidopsis* as well as *Lamium amplexicale* (*Lamium*) seeds were sown in a 6 cm diameter pot containing natural soil and stratified at 4 °C for 2 days. Pots were then transferred to the climate chamber and exposed to 12 h (light period)//12 h (dark period) under a light intensity of 200 µmol/m^2^ sec at 22 °C. The plants were maintained in the climate chamber for 4 weeks and irrigated twice per week. Thereafter, each plant was pulled out from the soil and gently shaken (to remove the aggregated bulk soil). The root (with remaining attached soil) was cut with a sterile scalpel from the aboveground organ, immediately snap-frozen, lyophilized for 3 days and lastly stored at −80 °C until further analysis.

### 3.3. Sample Preparation and Extraction Process

The dried root samples (~5 mg) were ground to a fine powder using a GenoGrinder 2010 from Spex (Metuchen, NJ, USA) for 1 min at 1500 RPM. The homogenized root samples were heated at 70 °C for 10 min in 1 mL of 70% aqueous methanol in a heating block to avoid myrosinase-mediated hydrolysis of glucosinolates according to a previously reported procedure [7,40]. The tubes were then vortexed, sonicated for 5 min, shaken at 30 RPM for 15 min in an Intelli-Mixer at 4 °C and centrifuged at 15,000× *g* in Sigma 1–14 K microcentrifuge (Buch and Holm, Herlev, Denmark). The supernatant was transferred into fresh tubes, diluted 1:1 and 1:50 (*v*/*v*) with 100% Milli-Q water, filtered through a 0.22-μm KX syringe filter (Mikrolab, Aarhus, Denmark) and injected into the LC-MS/MS system.

### 3.4. Tested Chromatographic Systems

The optimization tests were carried out on various reversed-phase high performance liquid chromatography (HPLC) analytical columns with different stationary phase compositions; Kinetex 2.6 µm XB-C_18_ 100 Å (100 × 2.1 mm), Synergi 4 µm Polar-RP 80 Å (250 × 2 mm) and Synergi 4 µm Fusion-RP, 80 Å column (250 mm × 2 mm) (all provided by Phenomenex). Four different mobile phases (with different solvent gradient programs, column oven temperatures and flow rates) with different modifiers were tested: (I) acetonitrile-water with 0.1% acetic acid (II) acetonitrile-water with 0.1% formic acid (III) methanol-water with 0.1% acetic acid (IV) methanol-water with 0.1% formic acid. The best result was obtained with the chromatographic conditions described in Section 3.5.

### 3.5. Final Recommended Method Used for Quantification of Intact Glucosinolates

The chromatographic system consisted of an Agilent 1260 infinity HPLC system (Santa Clara, CA, USA) coupled to an AB Sciex 4500 triple quadrupole-linear ion trap mass spectrometer (QTRAP/MS) (AB Sciex, Framingham, MA, USA). Data acquisition was performed using AB SCIEX Analyst 1.6.2 Software. The separation of intact glucosinolates was achieved using a Synergi Fusion-RP, 80A column (250 mm × 2 mm i.d., 4 μm, Phenomenex (S/N batch number: H16-143500 and B/N number: 5415-0061)) protected by a C18 Security Guard column (KJ0-4282, Phenomenex, Torrance, CA, USA). The injection volume was set to 10 µL and a binary solvent mixture was used with the gradient flow rate of 300 µL/min. The column compartment was maintained at 40 °C throughout the run. The LC conditions were as follows: Mobile phase “A” consisted of 100% water with 0.1% acetic acid and mobile phase “B” consisted of methanol with 0.1% acetic acid. The intact glucosinolates were separated by the following gradient: 0–3 min, column equilibration (95% A), 3–10 min, ramping to (80% A), 10–17 min, ramping to (55% A), 17–35 min, ramping to (0% A), 35–38 min, isocratic hold (0% A), 38–38.5 min, ramping back to (95% A), 38.5–45 min, returned to initial conditions for column re-equilibration (95% A).

The mass spectrometer was operated in negative ESI mode (for multiple reaction mode (MRM), precursor ion scans (Prec) and the enhanced product ion (EPI) scans). The instrument dependent source and gas parameters were determined for each analyte by flow injection analysis (FIA) and were as follows: curtain gas, 20 psi; ion spray voltage, −4000 V; ion source temperature, 550 °C; ion source gas 1 (nebulizer gas, ultra-high purity nitrogen), 70 psi; and ion source gas 2 (heater gas, ultra-high purity nitrogen), 40 psi. Nitrogen gas was used as a collision gas to generate MS/MS fragmentations. Compound dependent mass spectrometry parameters such as declustering potential (DP), entrance potential (EP), collision energy (CE) and collision cell exit potential (CXP) for MRM were determined by introducing individual reference standards to an electrospray source by direct infusion at the concentration level of 100 ng/mL in negative ESI mode. Accordingly, the fragments associated with the targeted deprotonated precursor molecule were determined. The MRM transition that corresponded to the highest signal was used for quantification and the other MRM transition was used for confirmation. The analyte identification was confirmed by comparing the retention time and the qualifier/quantifier ion ratio (with a tolerance of 20%) of the analytes in the sample with those obtained from the authentic analytical standard. A full list of transitions, DP, EP, CE and CXP can be found in Table 5.

Standard stock solutions (~1000 mg/L) of individual reference standards were prepared by dissolving each standard in either pure organic solvent (methanol or acetonitrile) or a mixture of organic solvent/milli-Q water and stored at −20 °C. A standard working solution (a mix of all standards) at a concentration of 10 mg/L was prepared in methanol and stored at −20 °C. Construction of the calibration curve and the processing of the acquired data from LC-MS/MS analysis were performed using Sciex vendor software, MultiQuant version 3.0.2. The 8-point calibration curve (over a concentration range from to 1.56 to 200 ng/mL) was constructed by plotting the ratios between the target analyte peak area and internal standard (sinalbin) peak area against the respective concentrations and the curve was fitted to a linear regression function with a weight of 1/x. The constructed calibration curve was employed for the quantitative determination of each target glucosinolate in the samples. Standard mixtures were analyzed in MRM mode. Each target glucosinolates were quantified using two MRM transitions (Table 5) and the average value was considered for absolute quantification.

### 3.6. Validation of Quantitation Method

Validation of the developed method was performed in accordance with Eurachem [17,41] and SANTE/11813/2017 [18] for eight glucosinolates. *Lamium* root was chosen as a blank matrix due to the similarity of its root texture to the *Arabidopsis* root as well as the absence of intact glucosinolates (confirmed by analyzing the methanolic root extract of Lamium by LC-MS/MS). Glucosinolate mixed standard solutions were spiked into the dried blank root samples with the purpose of obtaining the three concentration levels of a low, medium and high (3, 30 and 90 ng/mL) in the final extract. Six biological replicates were used per concentration. Known concentrations of analytes were spiked into the blank matrix, which then was allowed to equilibrate for 6 h at room temperature prior to extraction. The root samples were then ground to homogenous powder by using a mechanical disrupter Geno/Grinder 2010 from Spex (Metuchen, NJ, USA). The homogenized roots were extracted and analyzed with the LC-MS/MS method, as described in Section 3.5. This process was repeated at three concentrations levels over three consecutive days to test inter-day and on the same day for intra-day precision and accuracy. The accuracy was expressed as the mean relative error percentage (%RE) and was calculated with the following formula: (mean of concentration found − added concentration)/added concentration × 100 [42,43]. Precision was expressed as the relative standard deviation percentage (%RSD) and was calculated by dividing the standard deviation with the mean of detected concentration and multiplying by 100 [42]. Extraction efficiency (recovery) was calculated by dividing the mean peak area of glucosinolate standards spiked before extraction by the respective mean peak area of glucosinolate standards spiked to the blank matrix post-extraction and multiplying by 100 [42,44]. The matrix effect was calculated by comparing the mean peak area of the glucosinolates spiked into blank sample extract with peak areas of glucosinolates, which were prepared in the standard solvent. Limits of detection and quantification (LOD and LOQ) were determined by spiking blank samples with low concentrations of the standard compounds (the spiking concentrations were decided based on visual assessment prior to extraction) and calculating the LOD and LOQ as three and ten times the standard deviation of the concentration estimate of the spiked sample, respectively [45]. Linearity was determined using a matrix-matched calibration curve by spiking blank extract with analyte concentrations ranging from 1.56 to 200 ng/mL (a 8-point calibration curve), which was fitted by linear regression. Carry-over was evaluated by spiking *Lamium* root with the standard mixture at a concentration corresponding to the highest point of the calibration followed by injection of blank solvent in which the appearance of signals from the standards were controlled.

### 3.7. Prec97-IDA-EPI Method

A Precursor survey scan of all intact glucosinolate precursor ions that generated the common fragment ion *m*/*z* 97 was combined with the information-dependent acquisition (IDA) of the enhanced product ion (EPI) (Prec97-IDA-EPI) on the QqQ(LIT) instrument. Only precursors that generated the *m*/*z* fragment 97 were detected. Every time a precursor was detected above the predefined intensity threshold (2000 counts per second), an EPI spectral acquisition was triggered with which a full MS/MS fragmentation spectrum associated with the precursor peak was generated.

The exclusion time after the acquisition of the same ion was 5 s. The scan speed for Prec97 and EPI were 200 and 1000 Da/s, respectively. Fragments generated in the EPI scans were detected across a mass range of *m*/*z* 50–700 using normalized collision energy at −45 V with 15 V of collision energy spread (CES) (the spectra made with CE of −30, −45 and −60) and subsequently, a single spectrum was obtained by averaging the three scan results. The method was used for detection and subsequent structural characterization of individual glucosinolates of all structural classes in *Arabidopsis* roots.

### 3.8. Statistical Analysis

SIMCA-P (ver. 15.0.2, Umetrics AB, Umeå, Sweden) was used to perform principal component analysis (PCA). The ggplots2 package in R statistical language (version 3.5.3) was used to generate bar plot figures to observe variation in metabolite concentrations, which was expressed as means ± SEM (standard error of the mean).

## 4. Conclusions

An LC-MS/MS quantitation method, as well as an LC-QqQ(LIT) method for the identification of intact glucosinolates, were successfully developed. As part of the development of the LC-MS/MS method, the performance of three different reversed-phase chromatography columns was compared. The Synergi Fusion C18-based column was found to be effective for adequately retaining and separating intact glucosinolates without the need for ion-pairing reagents or a time-consuming sample preparation desulfation step. In particular, the established LC method was able to completely resolve the most polar structurally similar glucosinolates, 3msp and 4mtb, as well as the isobaric glucosinolates 4MOI3M and NMOI3M. Method validation using glucosinolate-free *Lamium* root as a blank matrix showed satisfactory inter-day and intra-day precision for all glucosinolates except for 4OHI3M. Full satisfactory inter-day and intra-day accuracy and extraction efficiency results were observed for all glucosinolates, except for 4OHI3M, 4msb and 4mtb, for which degradation likely occurred during the extraction process. Based on the acceptable %RSD of the recovery experiments correction of analytical results of samples in relation to recovery % can be done, in case true values are needed for a comparison with other studies. However, the use of an appropriate internal standard (if obtainable) for correcting for the loss of analyte during the sample treatment would be preferred in future studies. *Arabidopsis* root crude extract of 4 different accessions was analyzed with the established LC-MS/MS method and a natural variation in the glucosinolate composition and concentration was determined. The LC-QqQ(LIT) scanning method, Prec97-IDA-EPI, was found to be a novel approach for the screening of intact glucosinolate. The effectiveness of the proposed method was demonstrated in the analysis of crude root extracts of *Arabidopsis* accessions, where the Prec97-IDA-EPI scanning method enabled the simultaneous detection and structural characterization of a total of 20 intact glucosinolates from different structural classes. The Prec97-IDA-EPI approach proved to enhance the capacity of a triple quadrupole-linear ion trap instrument for the profiling of intact glucosinolate. This analytical approach has the potential to serve as a means for comprehensive detection and putative identification of a greater number of potential glucosinolates in other uncharacterized Brassica plant species.

## Figures and Tables

**Table 1:** Extraction efficiency and matrix effect of each analyte at three concentration levels tested (*n* = 6). Glucosinolate mixed standard solutions were spiked with the purpose of obtaining the three concentration levels of a low (3 ng/mL), medium (30 ng/mL) and high (90 ng/mL). Full names and abbreviations: glucoraphanin (4msb), glucoerucin (4mtb), glucoiberin (3msp), gluconapin (3but), glucobrassicin (I3M), 4-methoxyglucobrassicin (4MOI3M), neoglucobrassicin (NMOI3M) and 4-hydroxyglucobrassicin (4OHI3M).

Analyte	Mean Extraction Efficiency (%) ± RSD (%)			Mean Matrix Effect (%) ± RSD (%)		
	Low	Medium	High	Low	Medium	High
3msp	87 ± 5	91 ± 4	96 ± 4	116 ± 6	105 ± 5	106 ± 3
4msb	145 ± 7	137 ± 3	126 ± 4	123 ± 6	111 ± 7	107 ± 2
4mtb	36 ± 18	48 ± 8	65 ± 7	131 ± 4	113 ± 4	113 ± 3
3but	87 ± 7	91 ± 4	92 ± 4	128 ± 7	115 ± 7	113 ± 4
I3M	78 ± 10	85 ± 3	89 ± 4	136 ± 3	115 ± 4	113 ± 4
4MOI3M	78 ± 8	83 ± 4	86 ± 7	132 ± 2	116 ± 4	117 ± 2
NMOI3M	81 ± 7	84 ± 5	90 ± 4	127 ± 2	107 ± 1	115 ± 3
4OHI3M	46 ± 13	45 ± 12	41 ± 20	125 ± 13	123 ± 12	111 ± 7

**Table 2:** Intra-day and inter-day precision and accuracy of intact glucosinolates (*n* = 6). Glucosinolate mixed standard solutions were spiked into the blank root matrix of *Lamium* with the purpose of obtaining the three concentration levels of a low, medium and high (3, 30 and 90 ng/mL) in the final extract. Full names and abbreviations: glucoraphanin (4msb), glucoerucin (4mtb), glucoiberin (3msp), gluconapin (3but), glucobrassicin (I3M), 4-methoxyglucobrassicin (4MOI3M), neoglucobrassicin (NMOI3M) and 4-hydroxyglucobrassicin (4OHI3M). RSD: relative standard deviation; RE: relative error.

Validation Parameter	Spiked Conc. (ng/mL)	3msp	4msb	4mtb	3but	I3M	4MOI3M	NMOI3M	4OHI3M
Intra-day precision (%RSD)	3	9	5	4	4	4	15	6	17
	30	2	1	10	2	5	8	5	16
	90	6	3	11	2	3	12	3	21
Inter-day precision (%RSD)	3	10	10	9	2	7	6	8	22
	30	4	4	10	3	3	4	5	32
	90	7	9	9	2	6	6	8	27
Intra-day accuracy (%RE)	3	−10	53	−75	−20	−14	−28	−16	−60
	30	−3	48	−55	−10	−14	−20	−6	−70
	90	−2	21	−30	−8	−9	−28	−4	−72
Inter-day accuracy (%RE)	3	−8	56	−62	−5	−7	−12	−11	−47
	30	−7	34	−48	−4	−19	−20	−16	−65
	90	−5	29	−39	−5	−11	−17	−11	−67

**Table 3:** Limit of detection (LOD), limit of quantification (LOQ) and coefficient of determination (R^2^) values.

Validation Parameter	3msp	4msb	4mtb	3but	I3M	4MOI3M	NMOI3M	4OHI3M
LOD (µg/g)	0.05	0.19	0.05	0.06	0.06	0.04	0.04	0.14
LOQ (µg/g)	0.15	0.62	0.16	0.21	0.19	0.13	0.14	0.46
R^2^	0.99	0.99	0.99	0.99	0.99	0.99	0.99	0.99

**Table 4:** Prec97-IDA-EPI (precursor ion survey scan of *m*/*z* 97 in combination with the information-dependent acquisition (IDA) of the enhanced product ion (EPI) dependent scan) results where 20 intact glucosinolates were tentatively identified in the root of *Arabidopsis* accessions grown in natural soil.

Name	Abbrev.	Class	Ret. Time (min)	[M − H]^−^ (*m*/*z*)	MS/MS Product Ions *m*/*z* (Base Ion in Bold)
3-Hydroxypropyl	3ohp	hydroxyalkyl	4.2	376	75, 80, **97**, 134, 180, 259, 275, 297
Glucoiberin	3msp	sulfinylalkyl	4.8	422	75, 80, 97, 162, 180, 196, 259, 275, 342, **358**, 407
Progoitrin	2OH-3but	hydroxyalkenyl	5.4	388	75, 80, **97**, 136, 146, 259, 275, 301, 308, 332
Glucoraphanin	4msb	sulfinylalkyl	5.6	436	75, 80, 97, 178, 186, 225, 244, 259, 275, **372**, 421
2-Hydroxy-4-pentenyl	2OH-4pent	hydroxyalkenyl	85	402	97, 136, 160, **259**, 275, 305, 322, 366, 384
5-Methylsulfinylpentyl	5msp	sulfinylalkyl	8.3	450	97, 192,259, 275, **386**, 395, 432, 435
Gluconapin	3but	alkenyl	10	372	75, 80, **97**, 130, 139, 179, 259, 275, 294, 335, 354
4-Hydroxyglucobrassicin	4OHI3M	Indole glucosinolate	12.9	463	75, **97**, 132, 160, 169, 221, 259, 267, 275,285, 383
6-Methylsulfinylhexyl	6msh	sulfinylalkyl	13.2	464	80, 97, 158, 190, 206, 222, 259, 275, **400**, 449
Glucoerucin	4mtb	thioalkyl	16.7	420	97, 178, 224, **259**, 275, 305, 340, 360, 384
7-Methylsulfinylheptyl	7msh	sulfinylalkyl	17.5	478	75, 80, 97, 172, 192, 220, 259, 275, **414**, 464
Glucobrassicin	I3M	Indole glucosinolate	18.1	447	75, 80, **97**, 172, 205, 259, 275, 291, 367
8-Methylsulfonyloctyl	8msio	sulfonylalkyl	19.6	508	**97**, 250, 259, 275, 316, 363, 378, 444, 493
8-Methylsulfinyloctyl	8mso	sulfinylalkyl	20.9	492	75, 80, 97, 186, 235, 259, 275, 413, **428**, 477
4-Methoxyglucobrassicin	4MOI3M	Indole glucosinolate	22	477	75, 80, **97**, 137, 154, 202, 235, 259, 275, 292, 300
Neoglucobrassicin	NMOI3M	Indole glucosinolate	24.3	477	75, 80, **97**,154, 259, 275, 283, 365, 383, 446, 462
Glucomalcomiin	3bzo	benzoyloxy alkyl	24.6	480	75, 80, 97,121, 180, **196**, 241, 259, 275, 284, 358
6-Methylthiohexyl	6mth	thioalkyl	24.7	448	75, **97**, 206, 259, 270, 275, 368
7-Methylthioheptyl	7mth	thioalkyl	28.3	462	75, **97**, 139, 220, 259, 275, 340
8-Methylthiooctyl	8mto	thioalkyl	31.7	476	75, 80, **97**, 139, 163, 219, 227, 259, 275

**Table 5:** A complete list of all multiple reaction monitoring (MRM) transitions and compound-dependent parameters for liquid chromatography-electrospray ionization-mass spectrometry (LC-ESI-MS) of intact glucosinolates in negative-mode together with retention times for reference analytes and internal standard (Sinalbin). Retention time (Ret. Time), deprotonated molecule (Q1), product ion (Q3), declustering potential (DP), entrance potential (EP), collision energy (CE), collision cell exit potential (CXP). Each analyte has two MRM transitions.

Analyte	Abbrev.	Ret. Time (min)	Q1 (*m*/*z*)	Q3 (*m*/*z*)	DP (v)	EP (v)	CE (v)	CXP (v)
Glucoraphanin	4msb	5.8	436.1	95.8	−95	−7	−92	−13
			436.1	372.1	−95	−7	−28	−23
Glucoerucin	4mtb	16.7	420	95.8	−115	−12	−86	−9
			420	178	−115	−12	−36	0
Glucoiberin	3msp	4.8	422	95.8	−120	−5.5	−78	−11
			422	357.9	−120	−5.5	−28	−15
Gluconapin	3but	10	372.1	95.8	−60	−5.5	−54	−13
			372.1	74.8	−60	−5.5	−68	−5
Glucobrassicin	I3M	18.2	446.9	97	−85	−7	−54	−9
			446.9	259	−85	−7	−34	−17
Neoglucobrassicin	NMOI3M	24.3	477	96.8	−100	−6	−54	−15
			477	446	−100	−6	−22	−17
4-Methoxyglucobrassicin	4MOI3M	22	477	96.8	−100	−8.5	−84	−13
			477	74.9	−100	−8.5	−60	−9
4-hydoroxyglucobrassicin	4OHI3M	13	463	96.8	−105	−6	−50	−11
			463	74.9	−105	−6	−76	−15
Sinalbin	pOHB	9.1	423.9	95.8	−50	−7.5	−74	−15
			423.9	181.7	−50	−7.5	−32	−15

## Data Availability

The data presented in this study are available on request from the corresponding author or the first author. The data are not publicly available due to privacy.

## Supplementary Materials

The following are available online at https://www.mdpi.com/2218-1989/11/1/47/s1, Figure S1: MS/MS fragmentation spectra of naturally occurring intact glucosinolates in the root of *Arabidopsis* accessions by employing the prec97-IDA-EPI scanning method using LC-QqQ(LIT) mass spectrometry, Figure S2: LC–ESI-MS chromatograms of 9 individual glucosinolates (100 ng/mL standard mixture) using a Kinetex 2.6 µm XB-C18 (100 × 2.1 mm) column. The chromatographic and MS conditions are described in detail in Section 2.1 and Section 3.4 and Table 5, Figure S3: LC–ESI-MS chromatograms of 9 individual glucosinolates (100 ng/mL standard mixture) using a Synergi 4 µm Fusion-RP 80 Å (250 × 2 mm) (Phenomenex) analytical column with a C18 polar embedded functionality. The chromatographic conditions were as follows: (**A**) Mobile phase “A” consisted of 100% water with 0.1 acetic acid and mobile phase “B” consisted of methanol with 0.1% acetic acid. (**B**) Mobile phase “A” consisted of 100% water with 0.1% formic acid and mobile phase “B” consisted of methanol with 0.1% formic acid. (**C**) Mobile phase “A” consisted of 100% water with 0.1% acetic acid and mobile phase “B” consisted of acetonitrile with 0.1% acetic acid, Figure S4: LC–ESI-MS chromatograms of 9 individual glucosinolates (100 ng/mL standard mixture) using three different analytical columns. (**A**) Synergi 4 µm Fusion-RP C18 (250 × 2 mm). (**B**) Synergi 4 µm Polar-RP 80 Å (250 × 2 mm). (**C**) Kinetex 2.6 µm XB-C18 (100 × 2.1 mm). The mobile phase “A” consisted of 100% water with 0.1% acetic acid and mobile phase “B” consisted of methanol with 0.1% acetic acid, Figure S5: Calibration curves for each of 8 glucosinolates. The 8-point calibration curve (over a concentration range from 1.56 to 200 ng/mL) was constructed by plotting the ratios between the target analyte peak area and internal standard (sinalbin) peak area against the respective concentrations and the curve was fitted to a linear regression function with a weight of 1/x. The calibration curve was used for the quantitative determination of each target glucosinolate in the samples.

## References

[1] Halkier B.A., Gershenzon J. (2006). Biology and biochemistry of glucosinolates. Annu. Rev. Plant Biol..

[2] Grubb C.D., Abel S. (2006). Glucosinolate metabolism and its control. Trends Plant Sci..

[3] Sønderby I.E., Geu-Flores F., Halkier B.A. (2010). Biosynthesis of glucosinolates–gene discovery and beyond. Trends Plant Sci..

[4] Wink M. (2003). Evolution of secondary metabolites from an ecological and molecular phylogenetic perspective. Phytochemistry.

[5] Burow M., Halkier B.A., Kliebenstein D.J. (2010). Regulatory networks of glucosinolates shape Arabidopsis thaliana fitness. Curr. Opin. Plant Biol..

[6] Clarke D.B. (2010). Glucosinolates, structures and analysis in food. Anal. Methods.

[7] Crocoll C., Halkier B.A., Burow M. (2016). Analysis and quantification of glucosinolates. Curr. Protoc. Plant Biol..

[8] West L., Tsui I., Haas G. (2002). Single column approach for the liquid chromatographic separation of polar and non-polar glucosinolates from broccoli sprouts and seeds. J. Chromatogr. A.

[9] Ares A.M., Nozal M.J., Bernal J.L., Bernal J. (2014). Optimized extraction, separation and quantification of twelve intact glucosinolates in broccoli leaves. Food Chem..

[10] Clarke N.J., Rindgen D., Korfmacher W.A., Cox K.A. (2001). Peer Reviewed: Systematic LC/MS Metabolite Identification in Drug Discovery. Anal. Chem..

[11] Ji S., Wang Q., Qiao X., Guo H.-C., Yang Y.-F., Bo T., Xiang C., Guo D.-A., Ye M. (2014). New triterpene saponins from the roots of Glycyrrhiza yunnanensis and their rapid screening by LC/MS/MS. J. Pharm. Biomed..

[12] Qiao X., Lin X.-H., Ji S., Zhang Z.-X., Bo T., Guo D.-A., Ye M. (2016). Global profiling and novel structure discovery using multiple neutral loss/precursor ion scanning combined with substructure recognition and statistical analysis (MNPSS): Characterization of terpene-conjugated curcuminoids in Curcuma longa as a case study. Anal. Chem..

[13] Rochfort S.J., Trenerry V.C., Imsic M., Panozzo J., Jones R. (2008). Class targeted metabolomics: ESI ion trap screening methods for glucosinolates based on MSn fragmentation. Phytochemistry.

[14] Sánchez-Rabaneda F., Jáuregui O., Casals I., Andrés-Lacueva C., Izquierdo-Pulido M., Lamuela-Raventós R.M. (2003). Liquid chromatographic/electrospray ionization tandem mass spectrometric study of the phenolic composition of cocoa (*Theobroma cacao*). J. Mass Spectrom..

[15] Wu Z., Gao W., Phelps M.A., Wu D., Miller D.D., Dalton J.T. (2004). Favorable effects of weak acids on negative-ion electrospray ionization mass spectrometry. Anal. Chem..

[16] Snyder L.R., Dolan J.W. (2007). High-Performance Gradient Elution: The Practical Application of the Linear-Solvent-Strength Model.

[17] Magnusson B. (2014). The Fitness for Purpose of Analytical Methods: A Laboratory Guide to Method Validation and Related Topics (2014).

[18] Guidance Document on Analytical Quality Control and Method Validation Procedures for Pesticides Residues Analysis in Food and Feed. https://ec.europa.eu/food/sites/food/files/plant/docs/pesticides_mrl_guidelines_wrkdoc_2017-11813.pdf.

[19] Oerlemans K., Barrett D.M., Suades C.B., Verkerk R., Dekker M. (2006). Thermal degradation of glucosinolates in red cabbage. Food Chem..

[20] Jensen S.K., Liu Y.-G., Eggum B. (1995). The effect of heat treatment on glucosinolates and nutritional value of rapeseed meal in rats. Anim. Feed Sci. Technol..

[21] Hanschen F.S., Rohn S., Mewis I., Schreiner M., Kroh L.W. (2012). Influence of the chemical structure on the thermal degradation of the glucosinolates in broccoli sprouts. Food Chem..

[22] Ares A.M., Valverde S., Nozal M.J., Bernal J.L., Bernal J. (2016). Development and validation of a specific method to quantify intact glucosinolates in honey by LC–MS/MS. J. Food Compos. Anal..

[23] Thompson M., Ellison S.L., Wood R. (2002). Harmonized guidelines for single-laboratory validation of methods of analysis (IUPAC Technical Report). Pure Appl. Chem..

[24] Botting N.P., Robertson A.A., Morrison J.J. (2007). The synthesis of isotopically labelled glucosinolates for analysis and metabolic studies. J. Label. Compd. Radiopharm..

[25] Jeschke V., Kearney E.E., Schramm K., Kunert G., Shekhov A., Gershenzon J., Vassão D.G. (2017). How glucosinolates affect generalist lepidopteran larvae: Growth, development and glucosinolate metabolism. Front. Plant Sci..

[26] Schramm K., Vassão D.G., Reichelt M., Gershenzon J., Wittstock U. (2012). Metabolism of glucosinolate-derived isothiocyanates to glutathione conjugates in generalist lepidopteran herbivores. Insect Biochem. Mol. Biol..

[27] Kliebenstein D.J., Kroymann J., Brown P., Figuth A., Pedersen D., Gershenzon J., Mitchell-Olds T. (2001). Genetic control of natural variation in Arabidopsis glucosinolate accumulation. Plant Physiol..

[28] Witzel K., Hanschen F.S., Schreiner M., Krumbein A., Ruppel S., Grosch R. (2013). Verticillium suppression is associated with the glucosinolate composition of Arabidopsis thaliana leaves. PLoS ONE.

[29] Stotz H.U., Sawada Y., Shimada Y., Hirai M.Y., Sasaki E., Krischke M., Brown P.D., Saito K., Kamiya Y. (2011). Role of camalexin, indole glucosinolates, and side chain modification of glucosinolate-derived isothiocyanates in defense of Arabidopsis against Sclerotinia sclerotiorum. Plant J..

[30] Iven T., König S., Singh S., Braus-Stromeyer S.A., Bischoff M., Tietze L.F., Braus G.H., Lipka V., Feussner I., Dröge-Laser W. (2012). Transcriptional activation and production of tryptophan-derived secondary metabolites in Arabidopsis roots contributes to the defense against the fungal vascular pathogen Verticillium longisporum. Mol. Plant.

[31] Sarwar M., Kirkegaard J., Wong P., Desmarchelier J. (1998). Biofumigation potential of brassicas. Plant Soil.

[32] Tierens K.F.-J., Thomma B.P., Brouwer M., Schmidt J., Kistner K., Porzel A., Mauch-Mani B., Cammue B.P., Broekaert W.F. (2001). Study of the role of antimicrobial glucosinolate-derived isothiocyanates in resistance of Arabidopsis to microbial pathogens. Plant Physiol..

[33] Witzel K., Hanschen F.S., Klopsch R., Ruppel S., Schreiner M., Grosch R. (2015). Verticillium longisporum infection induces organ-specific glucosinolate degradation in Arabidopsis thaliana. Front. Plant Sci..

[34] Bressan M., Roncato M.-A., Bellvert F., Comte G., el Zahar Haichar F., Achouak W., Berge O. (2009). Exogenous glucosinolate produced by Arabidopsis thaliana has an impact on microbes in the rhizosphere and plant roots. ISME J..

[35] Zeng R.S., Mallik A.U., Setliff E. (2003). Growth stimulation of ectomycorrhizal fungi by root exudates of Brassicaceae plants: Role of degraded compounds of indole glucosinolates. J. Chem. Ecol..

[36] Burow M., Halkier B.A. (2017). How does a plant orchestrate defense in time and space? Using glucosinolates in Arabidopsis as case study. Curr. Opin. Plant Biol..

[37] Brown P.D., Tokuhisa J.G., Reichelt M., Gershenzon J. (2003). Variation of glucosinolate accumulation among different organs and developmental stages of Arabidopsis thaliana. Phytochemistry.

[38] Blazenovic I., Kind T., Ji J., Fiehn O. (2018). Software Tools and Approaches for Compound Identification of LC-MS/MS Data in Metabolomics. Metabolites.

[39] Fabre N., Poinsot V., Debrauwer L., Vigor C., Tulliez J., Fourasté I., Moulis C. (2007). Characterisation of glucosinolates using electrospray ion trap and electrospray quadrupole time-of-flight mass spectrometry. Phytochem. Anal..

[40] López-Berenguer C., Martínez-Ballesta M.C., García-Viguera C., Carvajal M. (2008). Leaf water balance mediated by aquaporins under salt stress and associated glucosinolate synthesis in broccoli. Plant Sci..

[41] Eurachem G. (1998). Harmonized, guidelines for the use of recovery information in analytical measurement. Pure Appl. Chem..

[42] DeFelice B.C., Fiehn O. (2019). Rapid LC-MS/MS quantification of cancer related acetylated polyamines in human biofluids. Talanta.

[43] Skoog D.A., West D.M., Holler F.J., Crouch S.R. (2013). Fundamentals of Analytical Chemistry.

[44] Matuszewski B., Constanzer M., Chavez-Eng C. (2003). Strategies for the assessment of matrix effect in quantitative bioanalytical methods based on HPLC−MS/MS. Anal. Chem..

[45] Hooshmand K., Kudjordjie E.N., Nicolaisen M., Fiehn O., Fomsgaard I.S. (2020). Mass Spectrometry-Based Metabolomics Reveals a Concurrent Action of Several Chemical Mechanisms in Arabidopsis-Fusarium oxysporum Compatible and Incompatible Interactions. J. Agric. Food Chem..

