# Supplementary material for: Analytical Methods for Quantification and Identification of Intact Glucosinolates in Arabidopsis Roots Using LC-QqQ(LIT)-MS/MS
Source: Metabolites. 2021 Jan 11;11(1):47. doi: 10.3390/metabo11010047 (PMC7826904; doi:10.3390/metabo11010047)

Supplementary information

Analytical Methods for Quantification and Identification of Intact Glucosinolates in Arabidopsis Roots Using LC-QqQ(LIT)-MS/MS

Kourosh Hooshmand, Inge S. Fomsgaard *
Department of Agroecology, Aarhus University, DK-4200 Slagelse, Denmark

* Correspondence: inge.fomsgaard@agro.au.dk; Tel.: +4587158212

Figure S1. MS/MS fragmentation spectra of 20 naturally occurring intact glucosinolates in the root of *Arabidopsis* accessions grown in the non-sterile natural soil by employing the prec97-IDA-EPI scanning method using LC-QqQ(LIT) mass spectrometry.

3-Hydroxypropyl glucosinolate (3ohp)

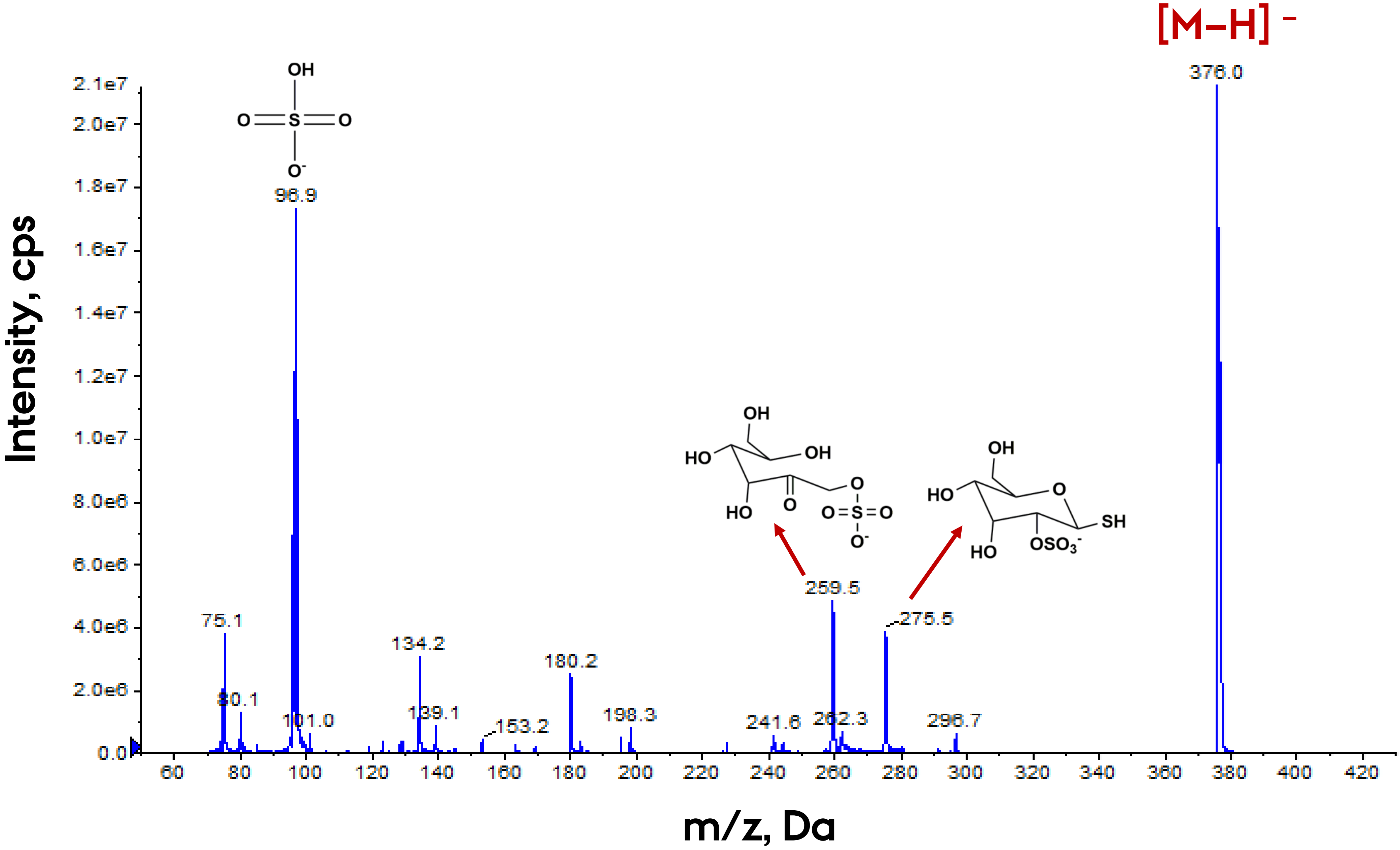

Glucoiberin (3msp)

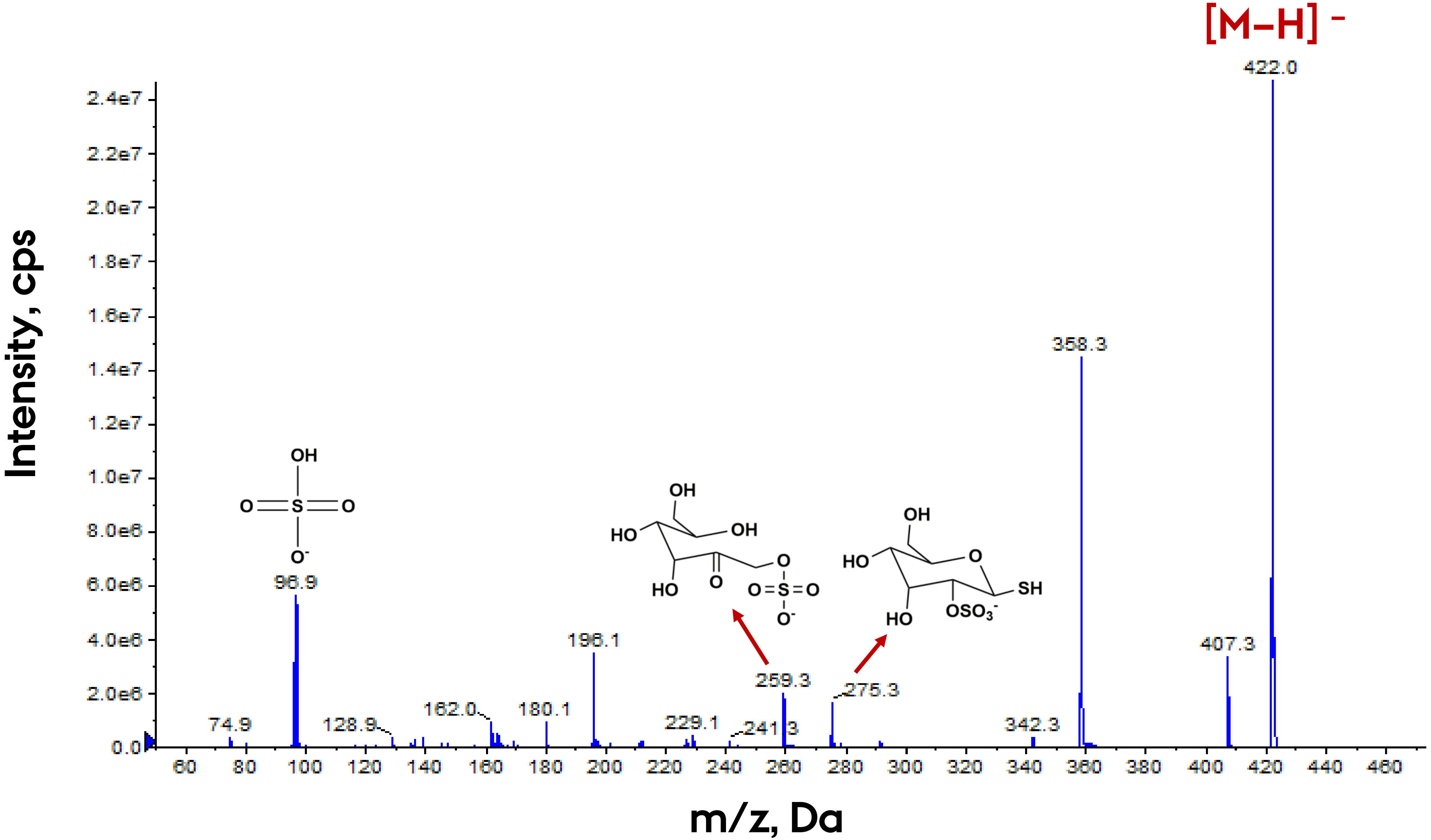

Progoitrin (2OH-3but)

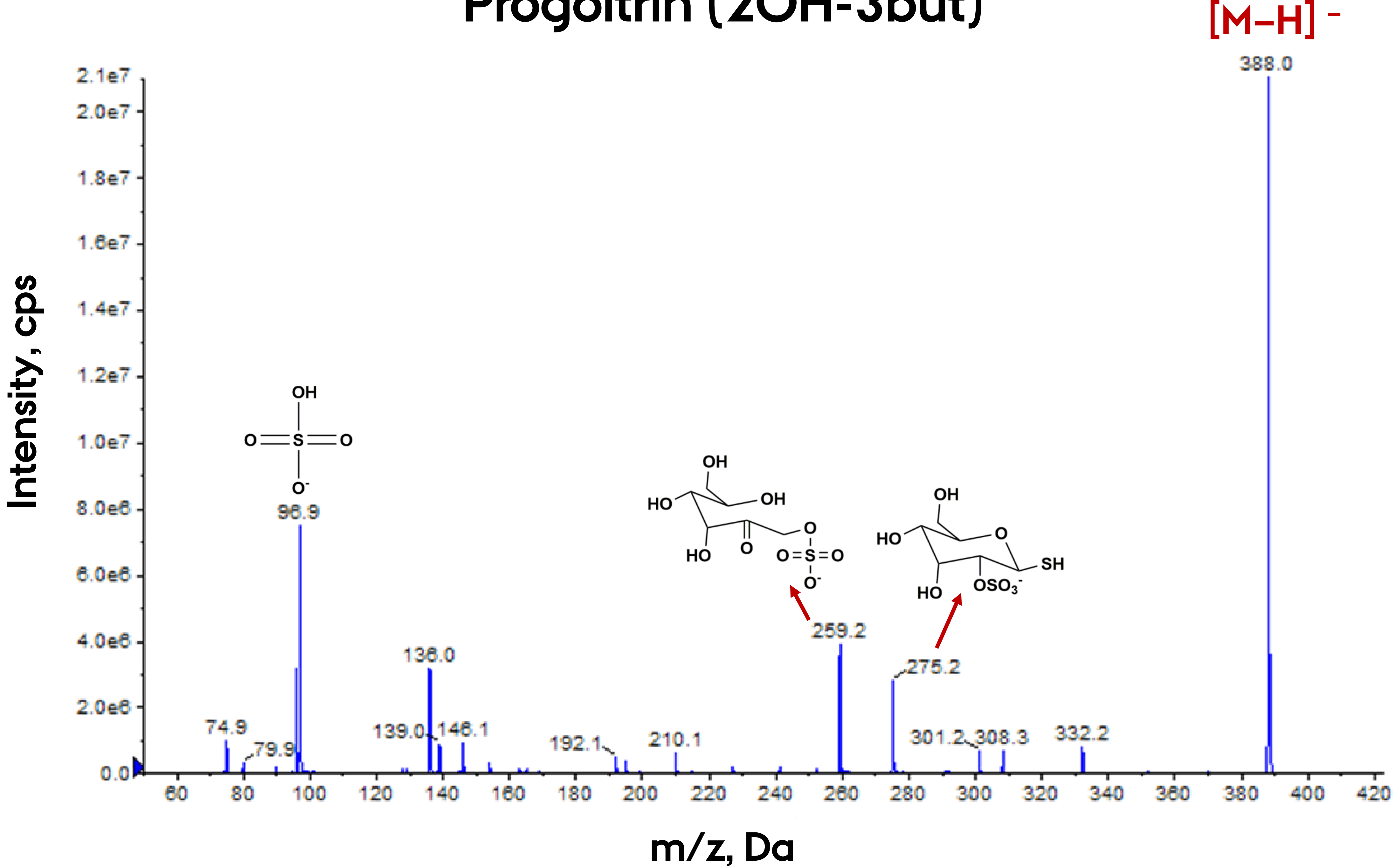

Glucoraphanin (4msb)

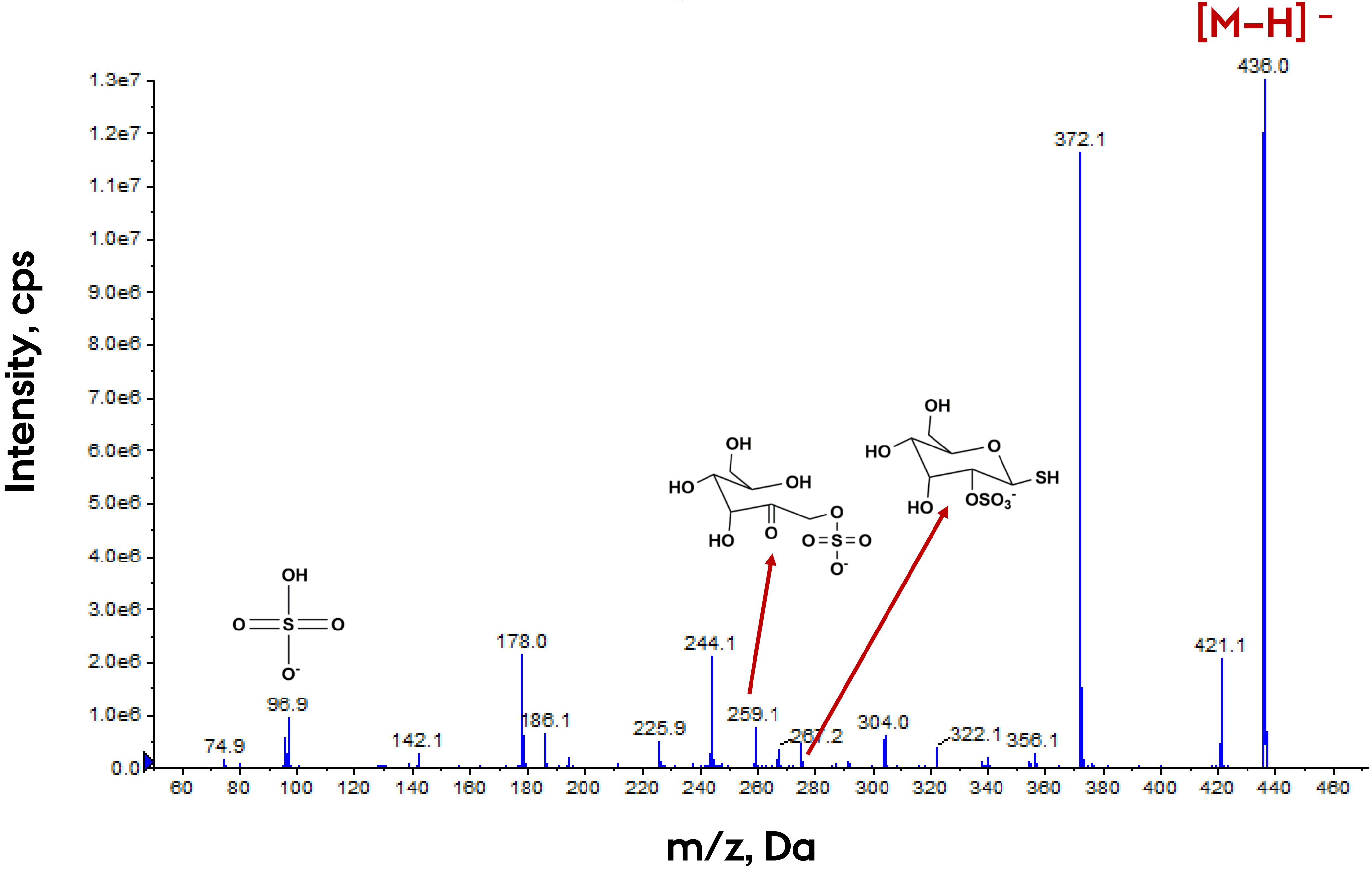

Gluconapoleiferin (2-OH-4-pent)

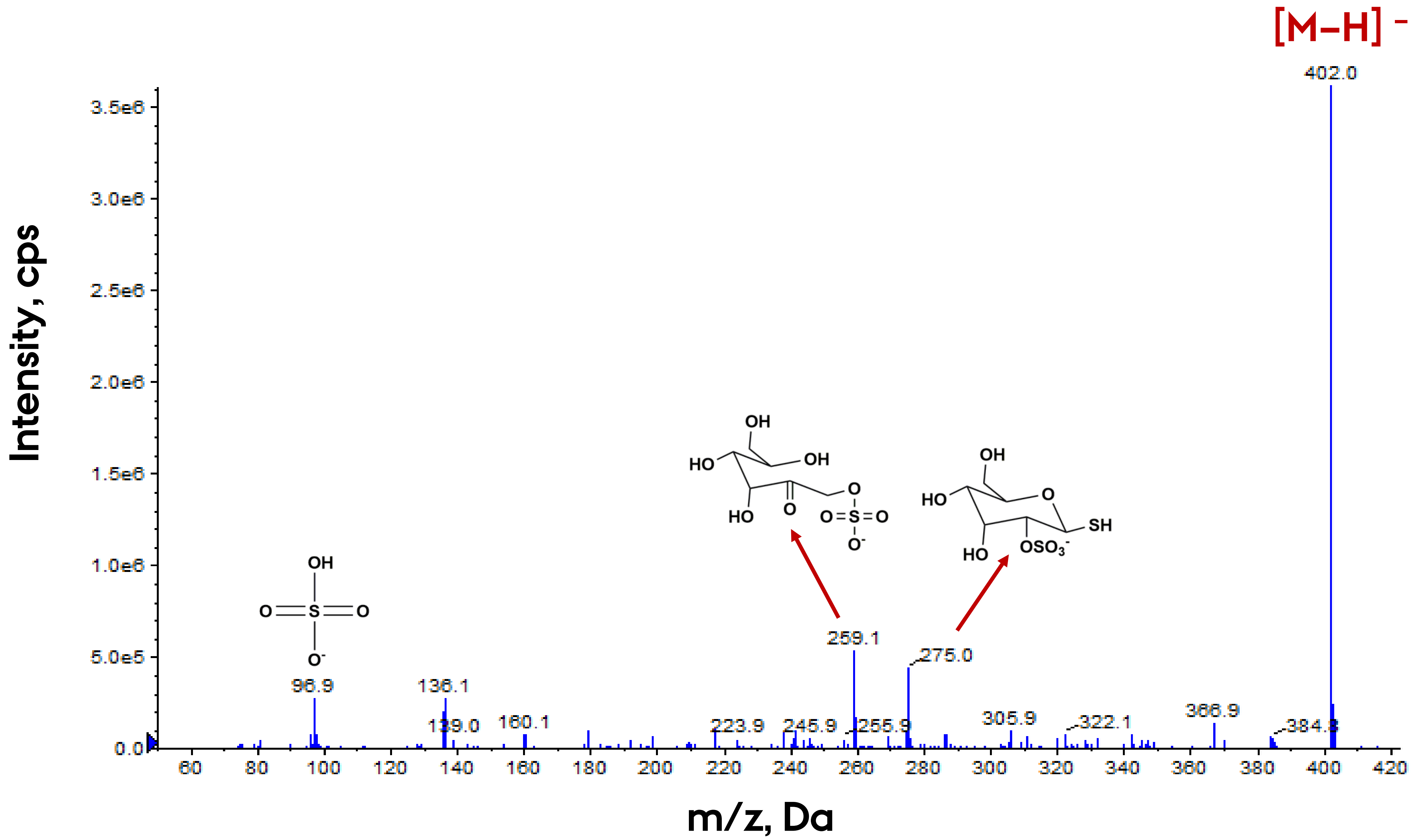

5-methylsulfinylpentyl glucosinolate (5msp)

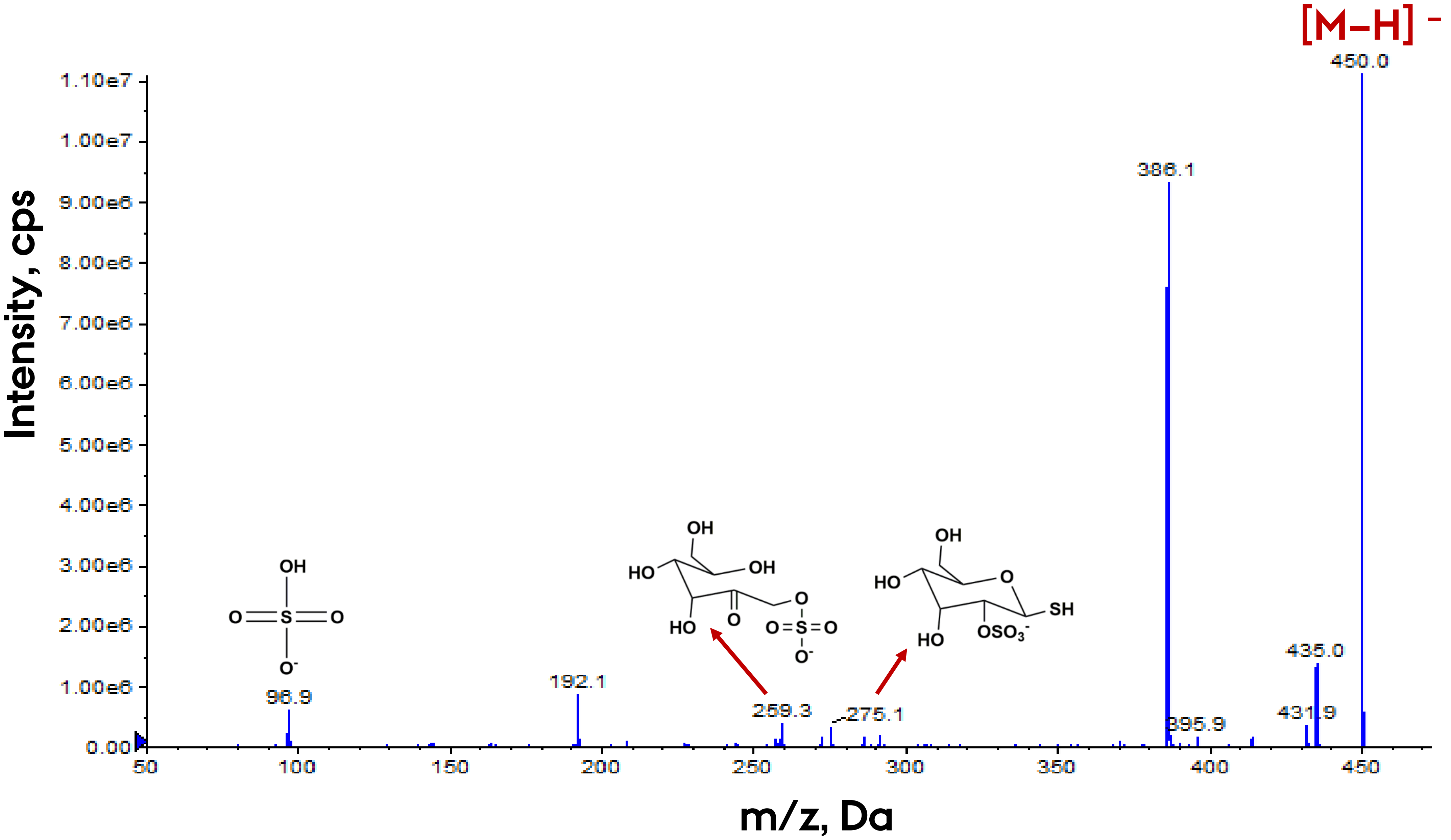

Gluconapin (3but)

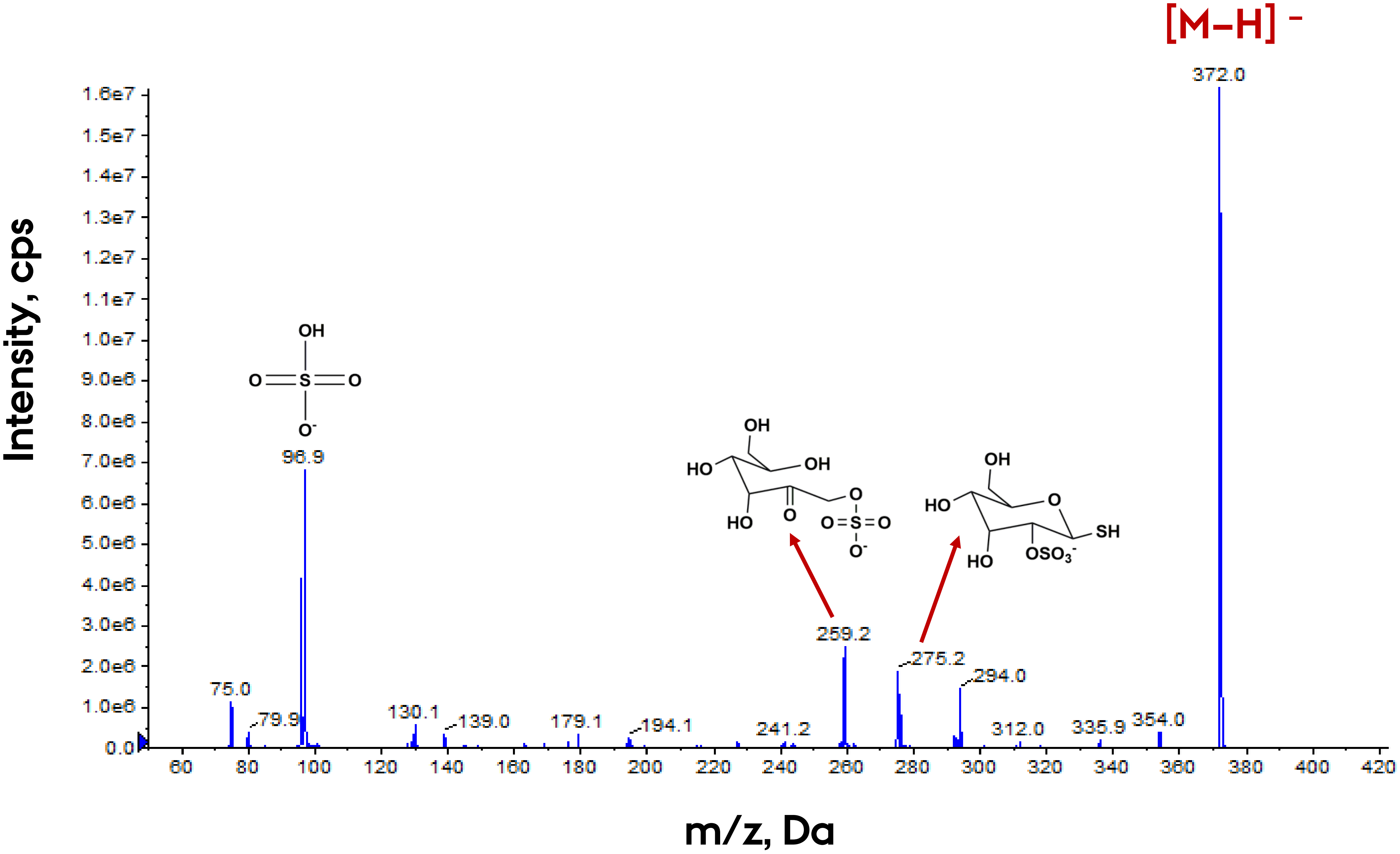

4-hydroxyglucobrassicin (4OHI3M)

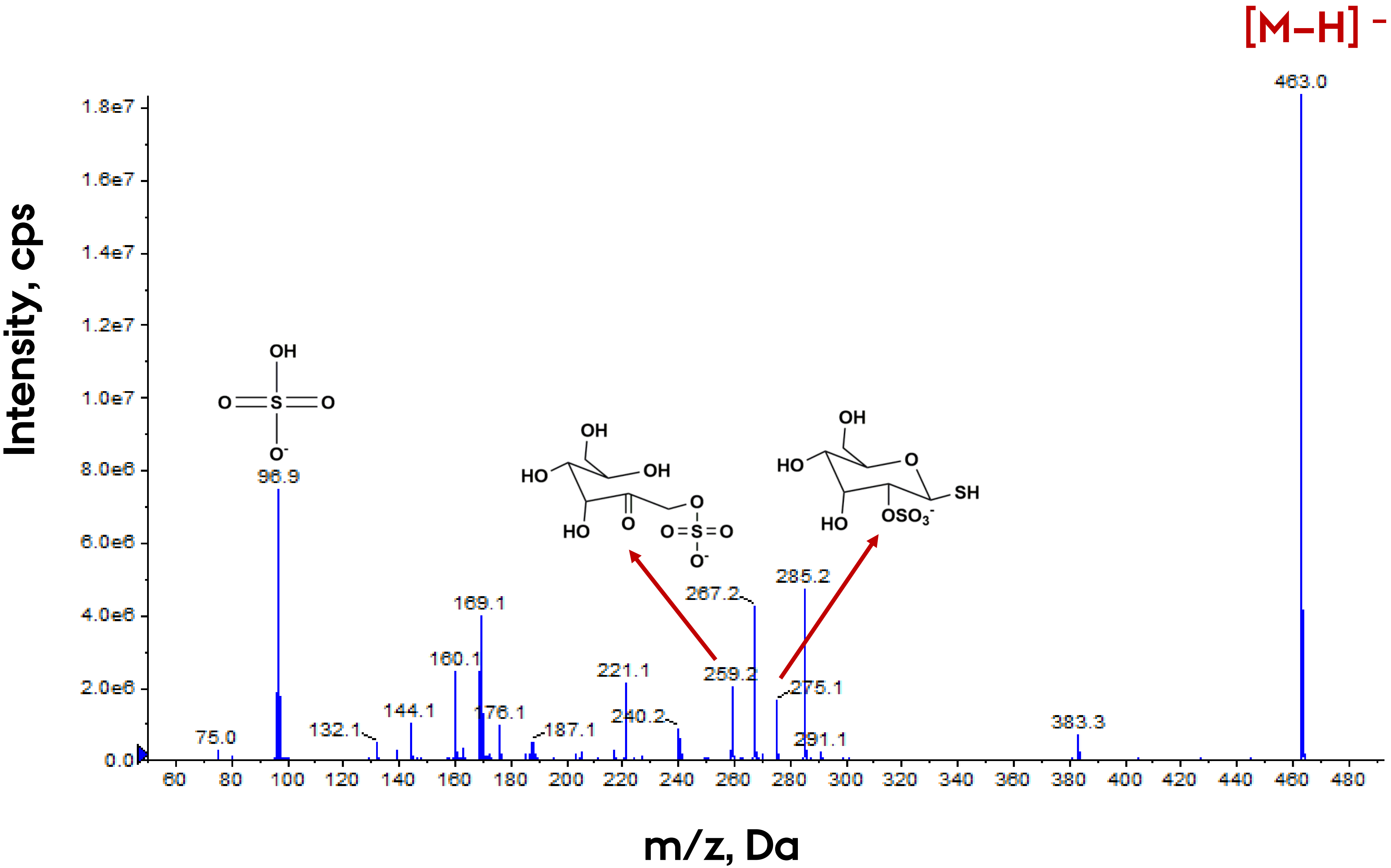

6-methylsulfinylhexyl or glucohesperin (6msh)

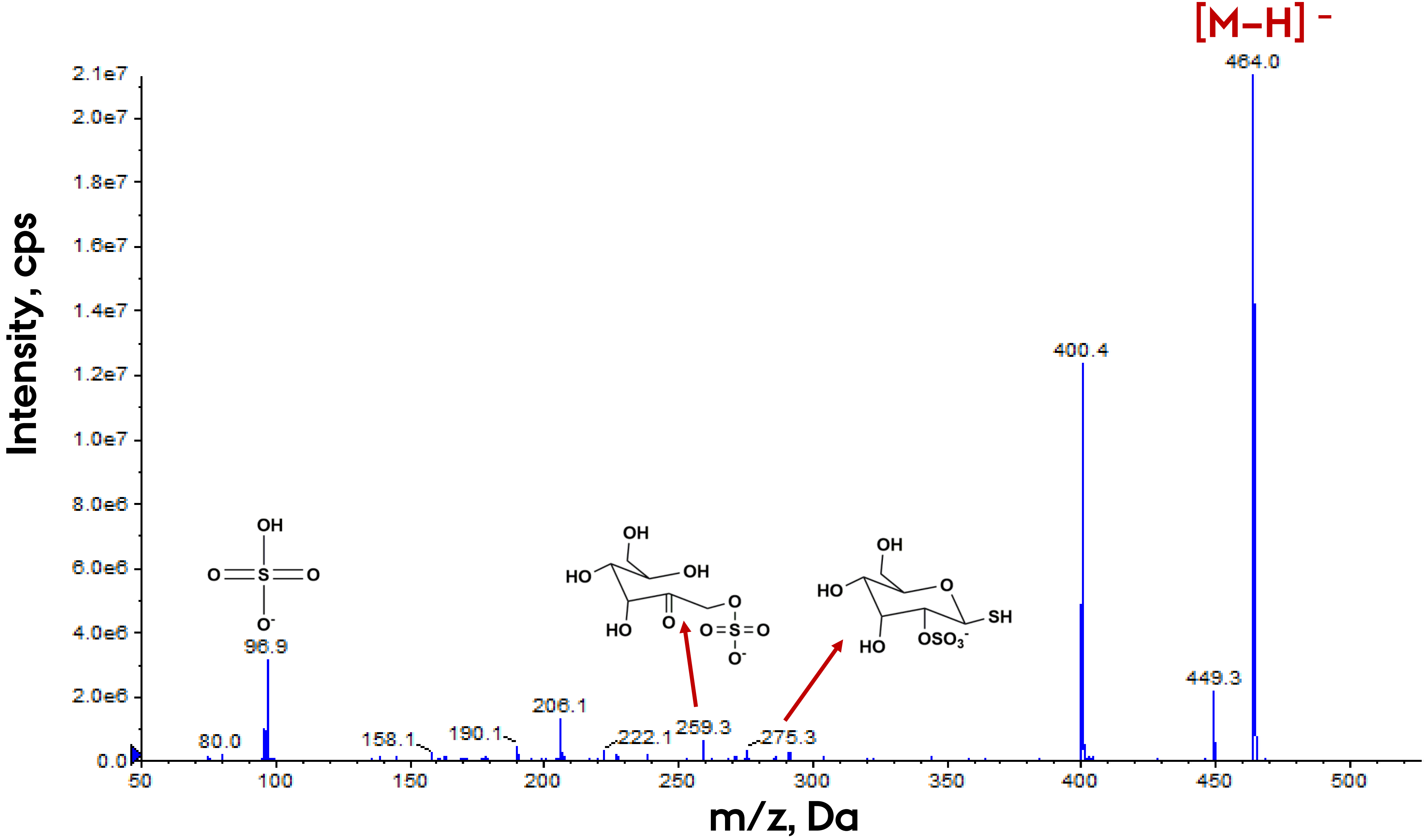

Glucoerucin (4mtb)

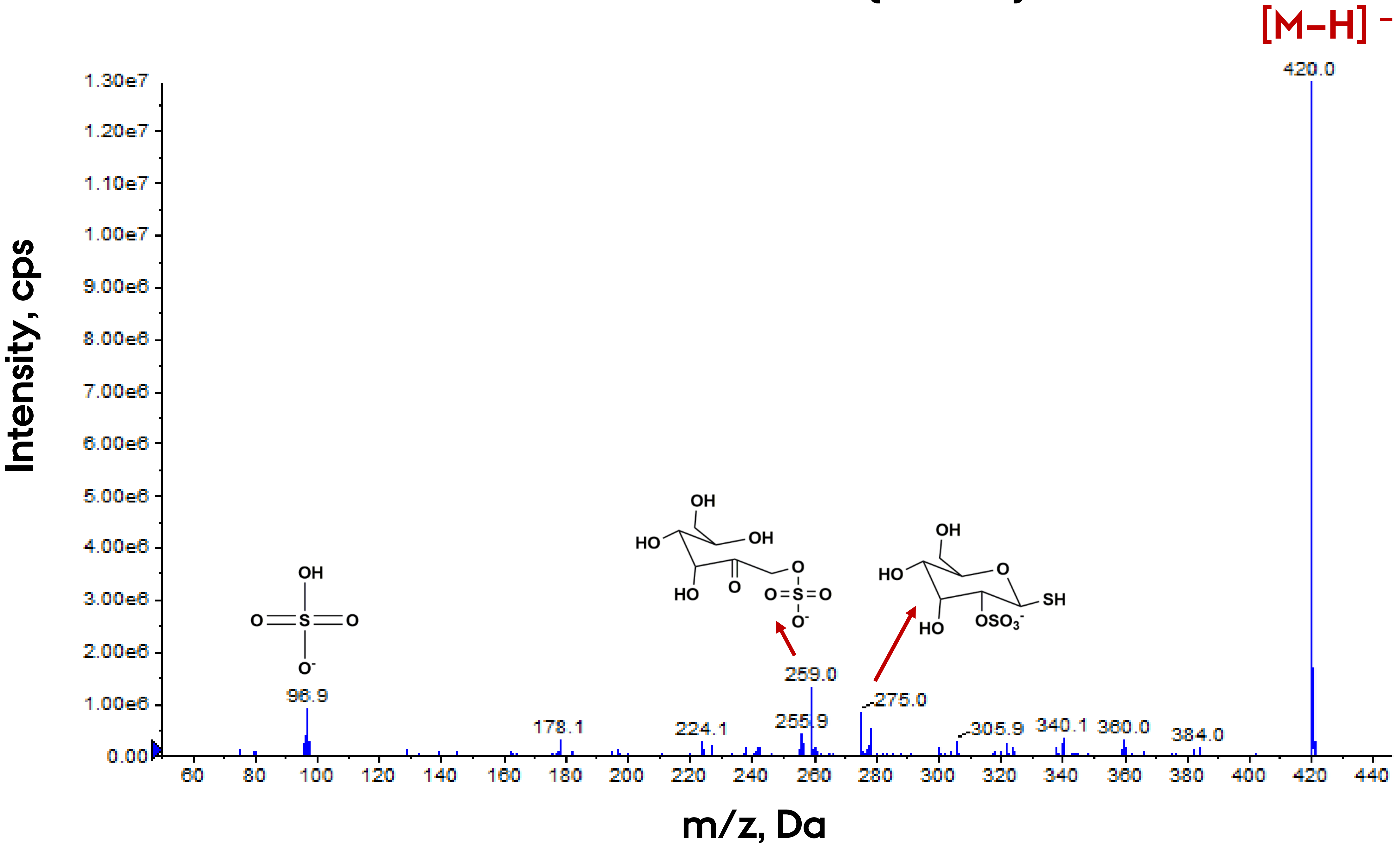

7-Methylsulfinylheptyl glucosinolate (or glucoibarin) (7msh)

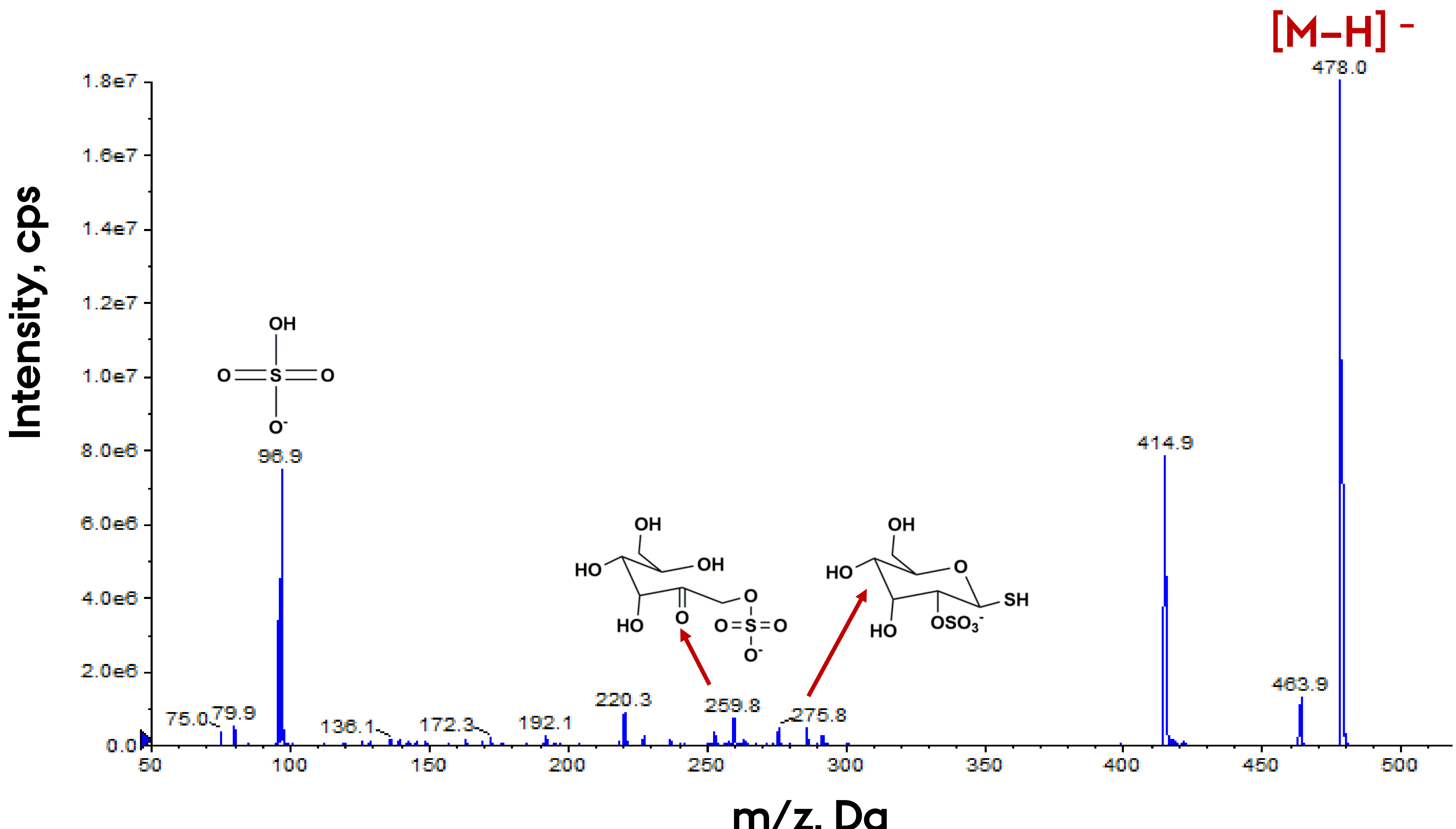

Glucobrassicin (I3M)

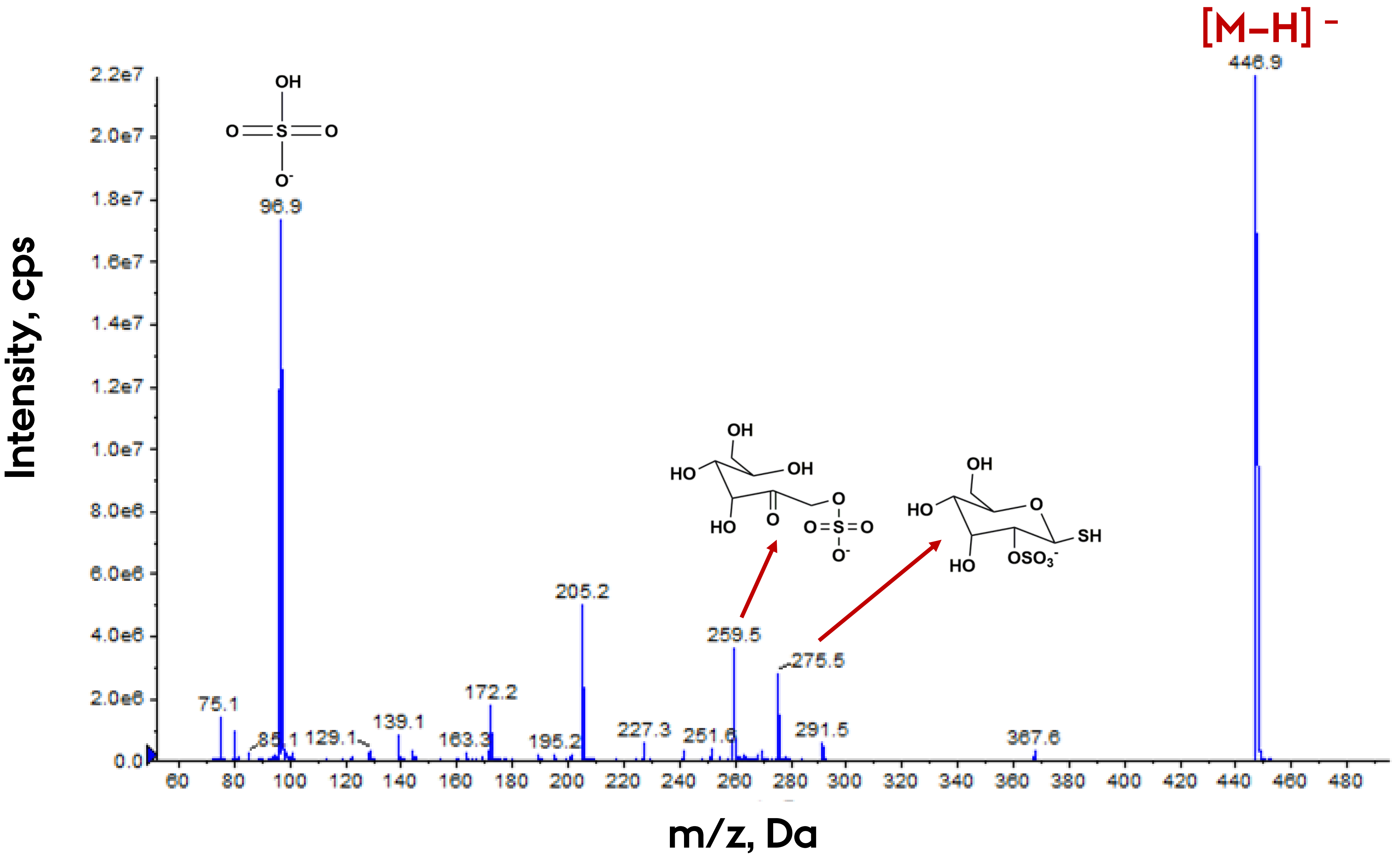

8-(methylsulfonyl) octyl glucosinolate (8msio)

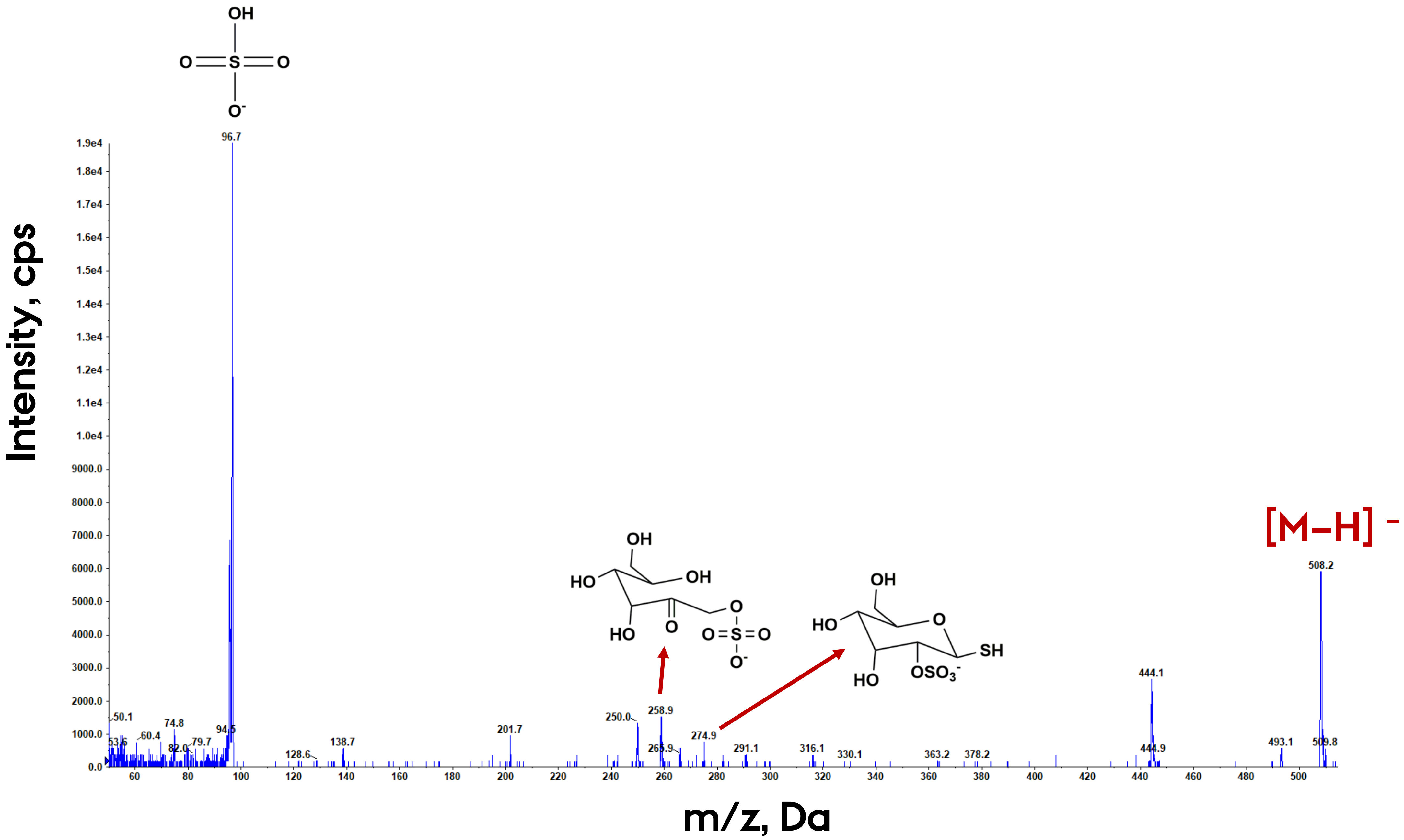

8-Methylsulfinyloctyl glucosinolate (or glucohirsutin) (8mso)

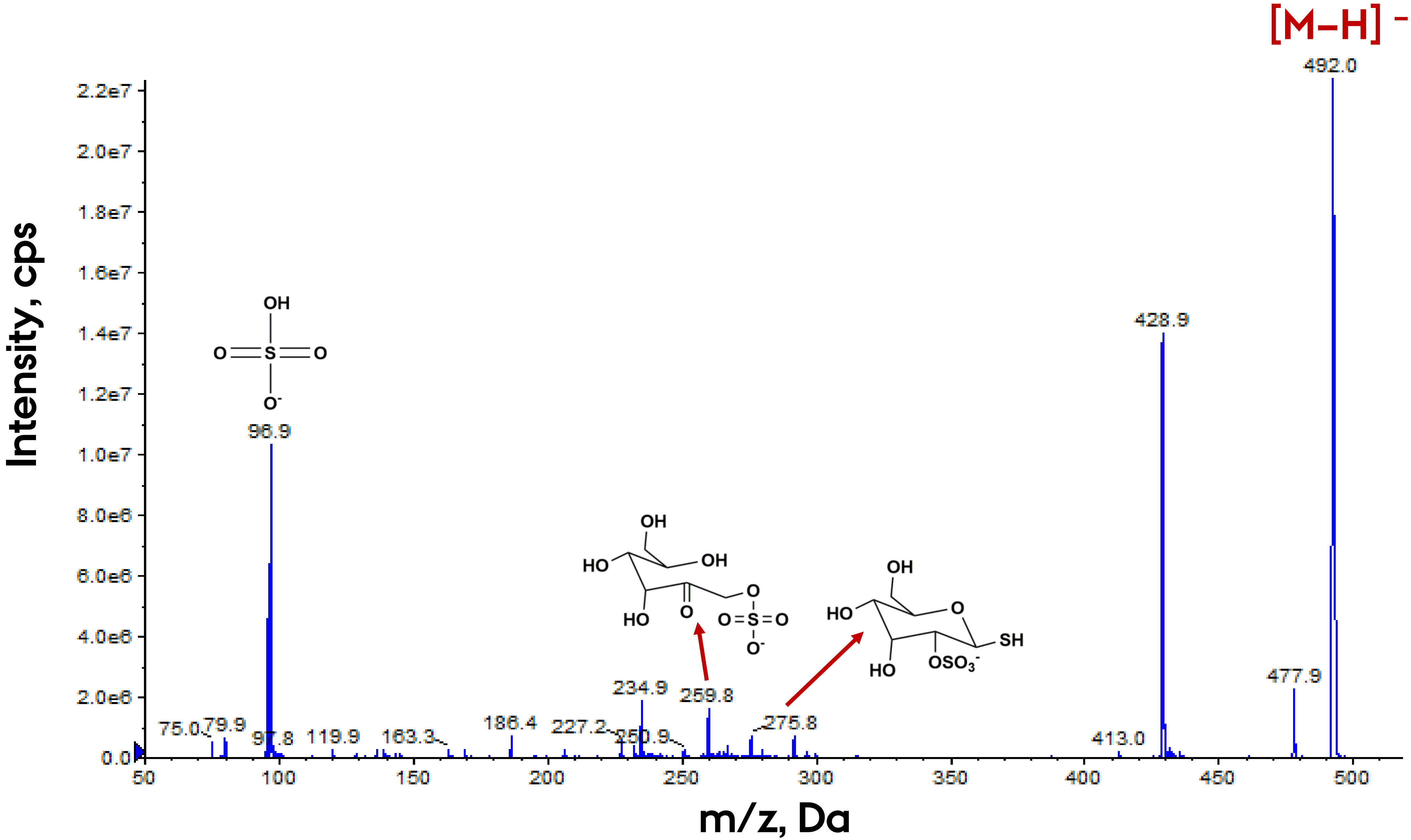

4-Methoxyglucobrassicin (4MOI3M)

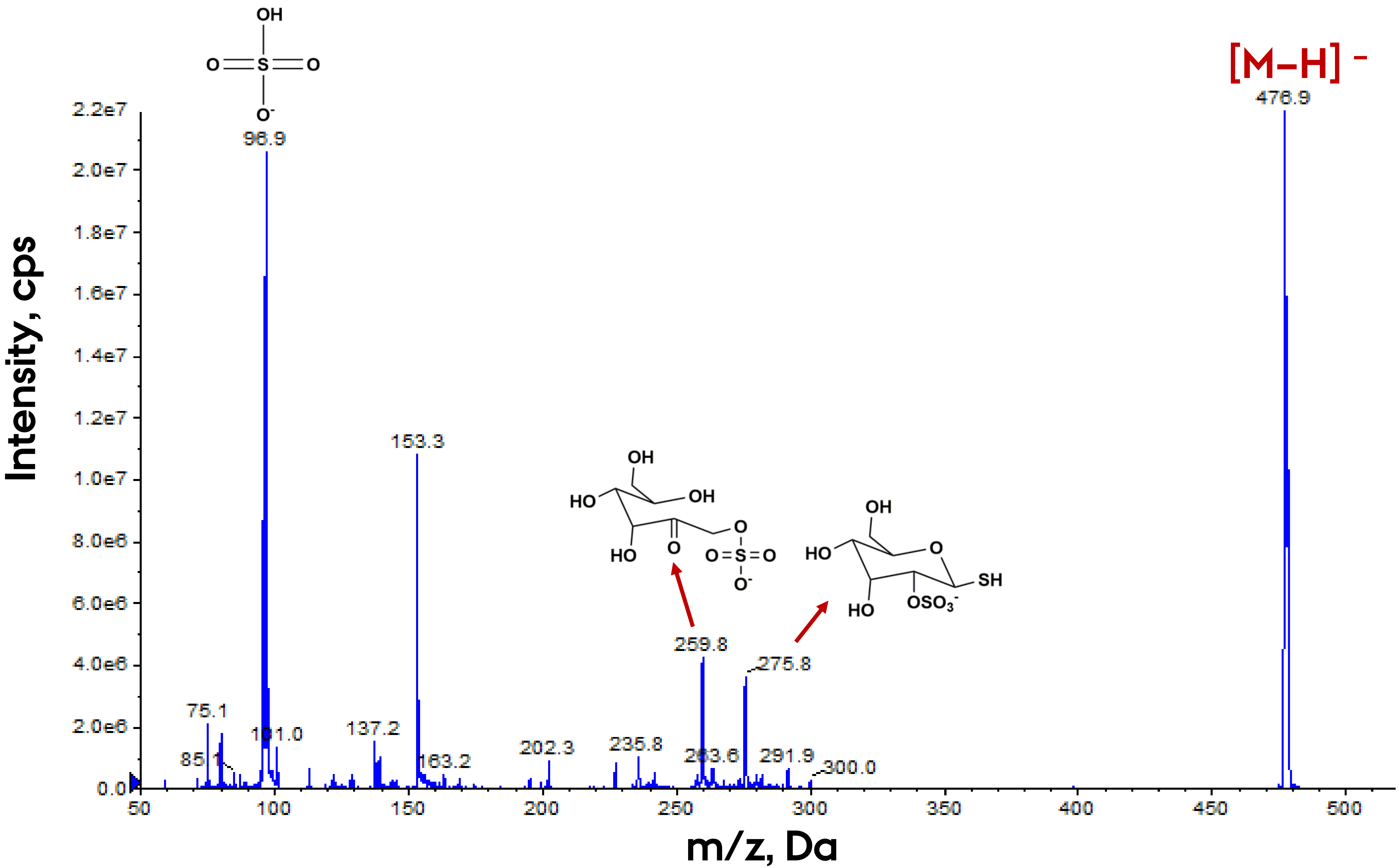

Neoglucobrassicin (NMOI3M)

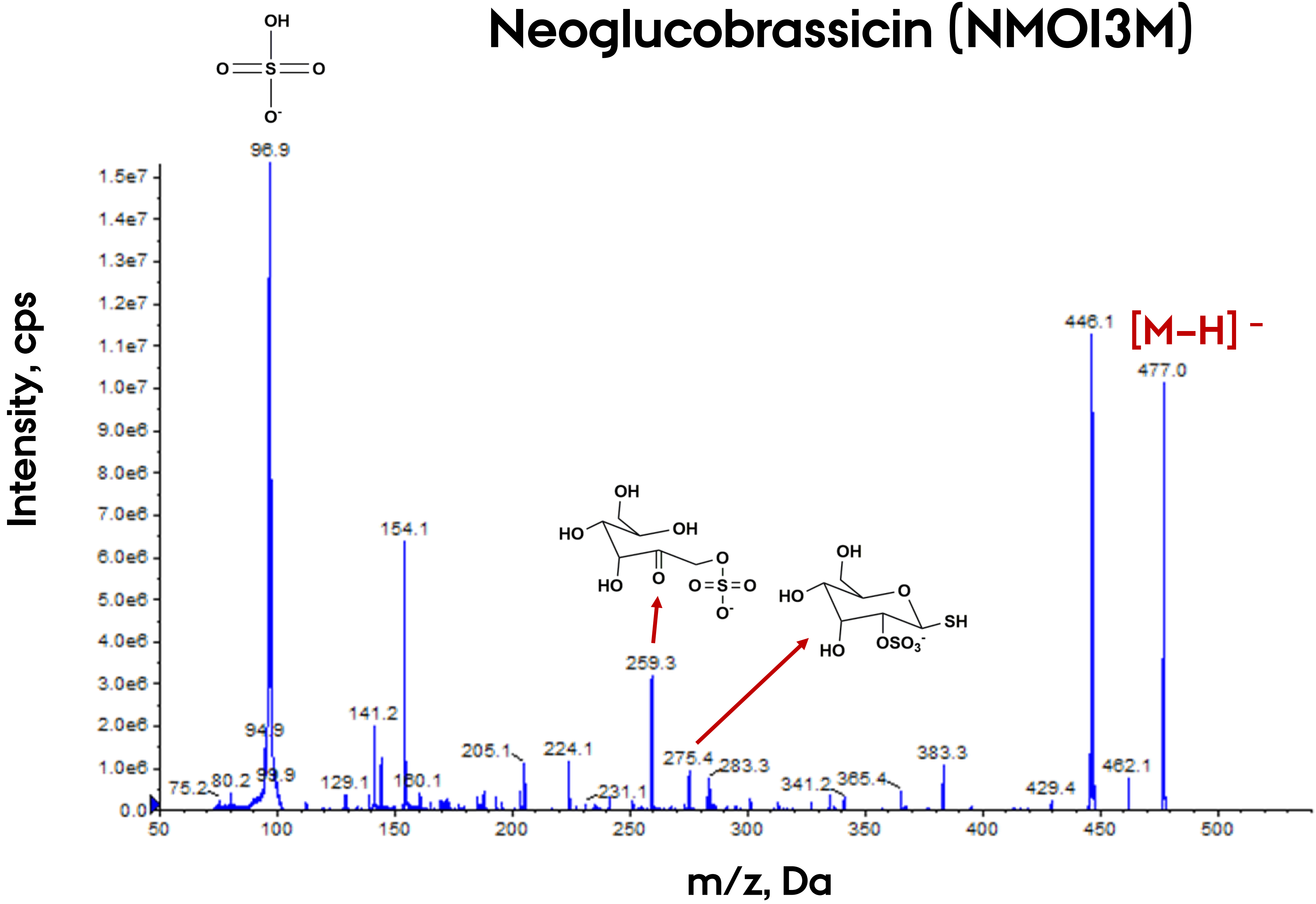

Glucomalcomiin (3bzo)

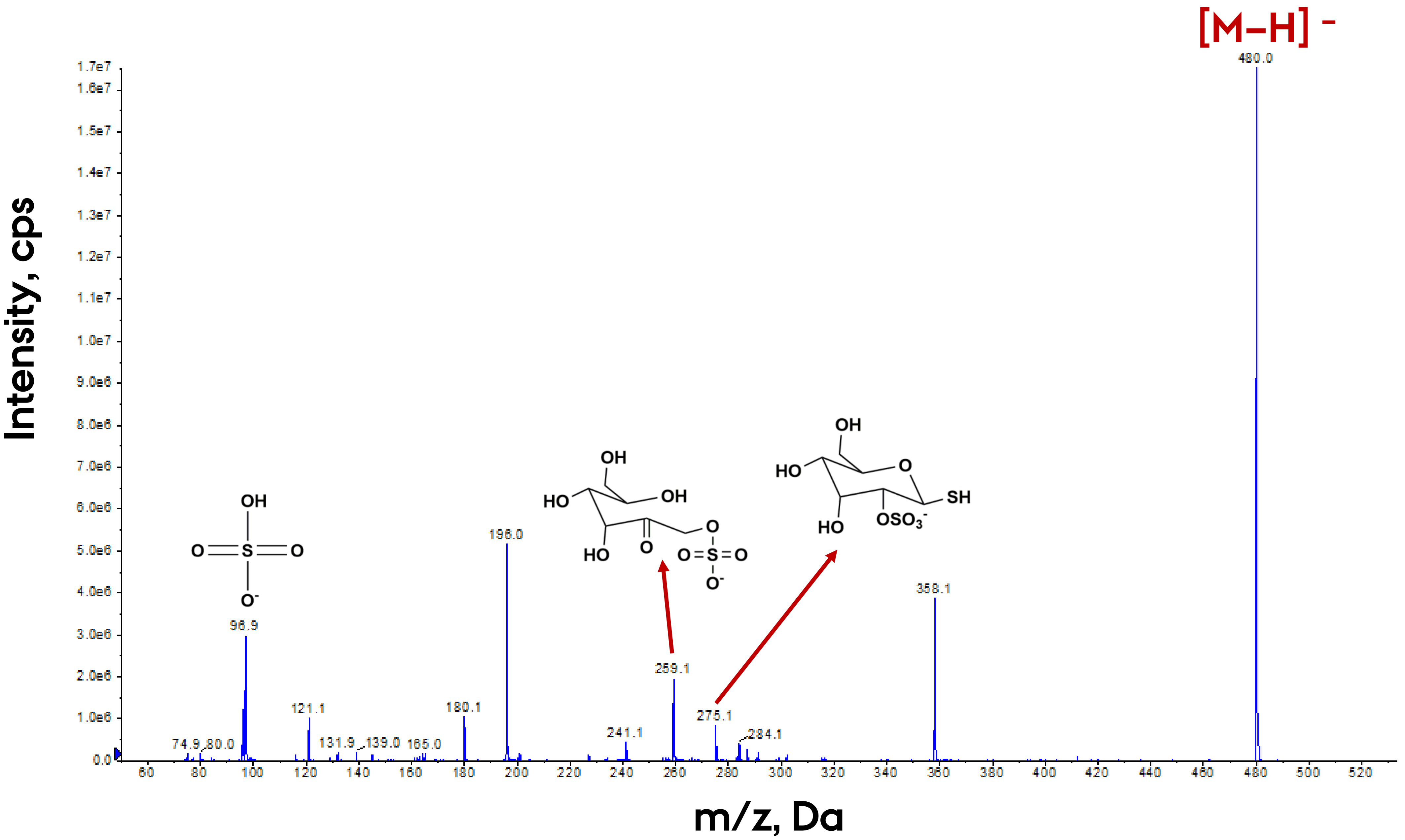

Glucolesquerellin (6mth)

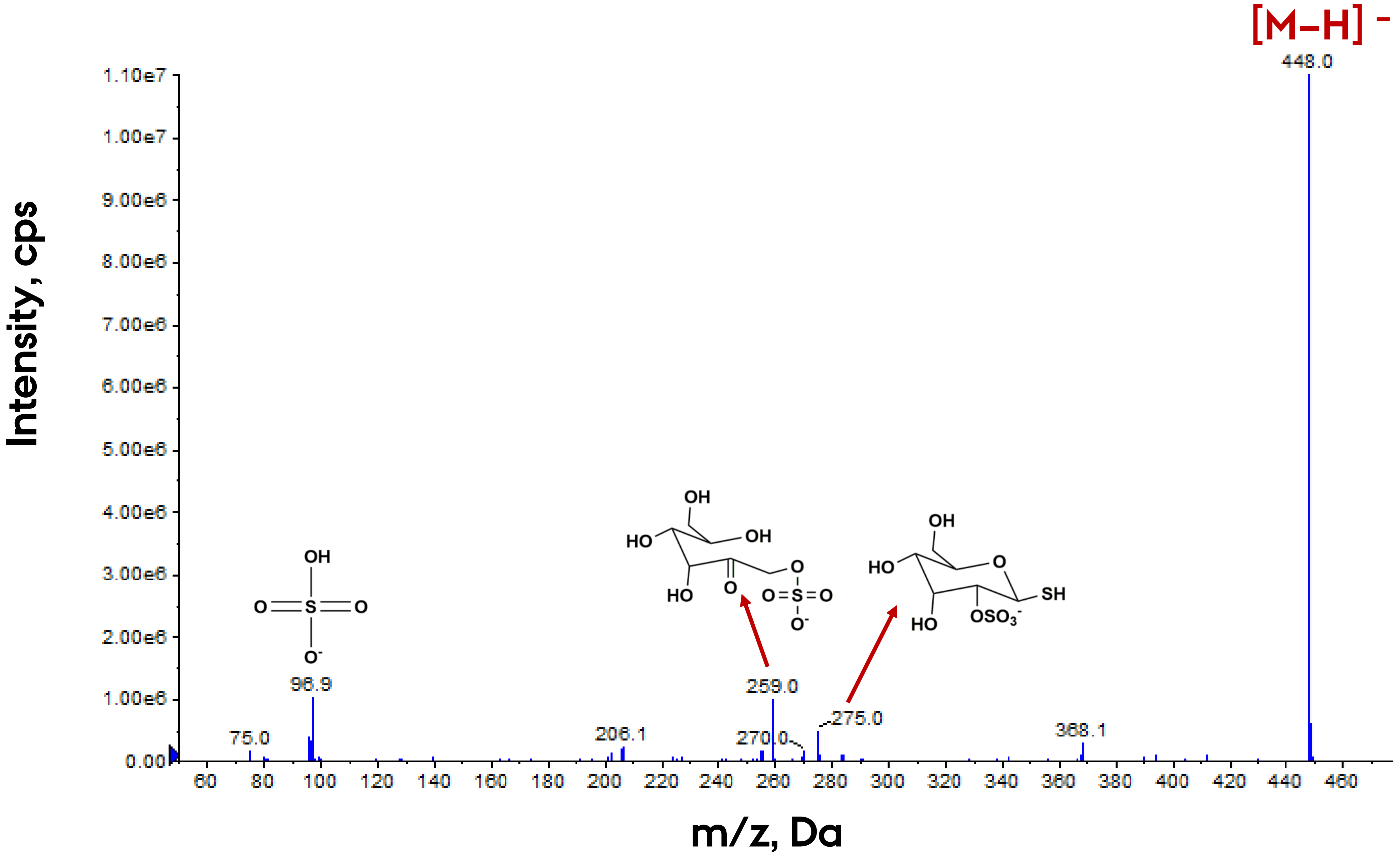

7-methylthioheptyl glucosinolate (7mth)

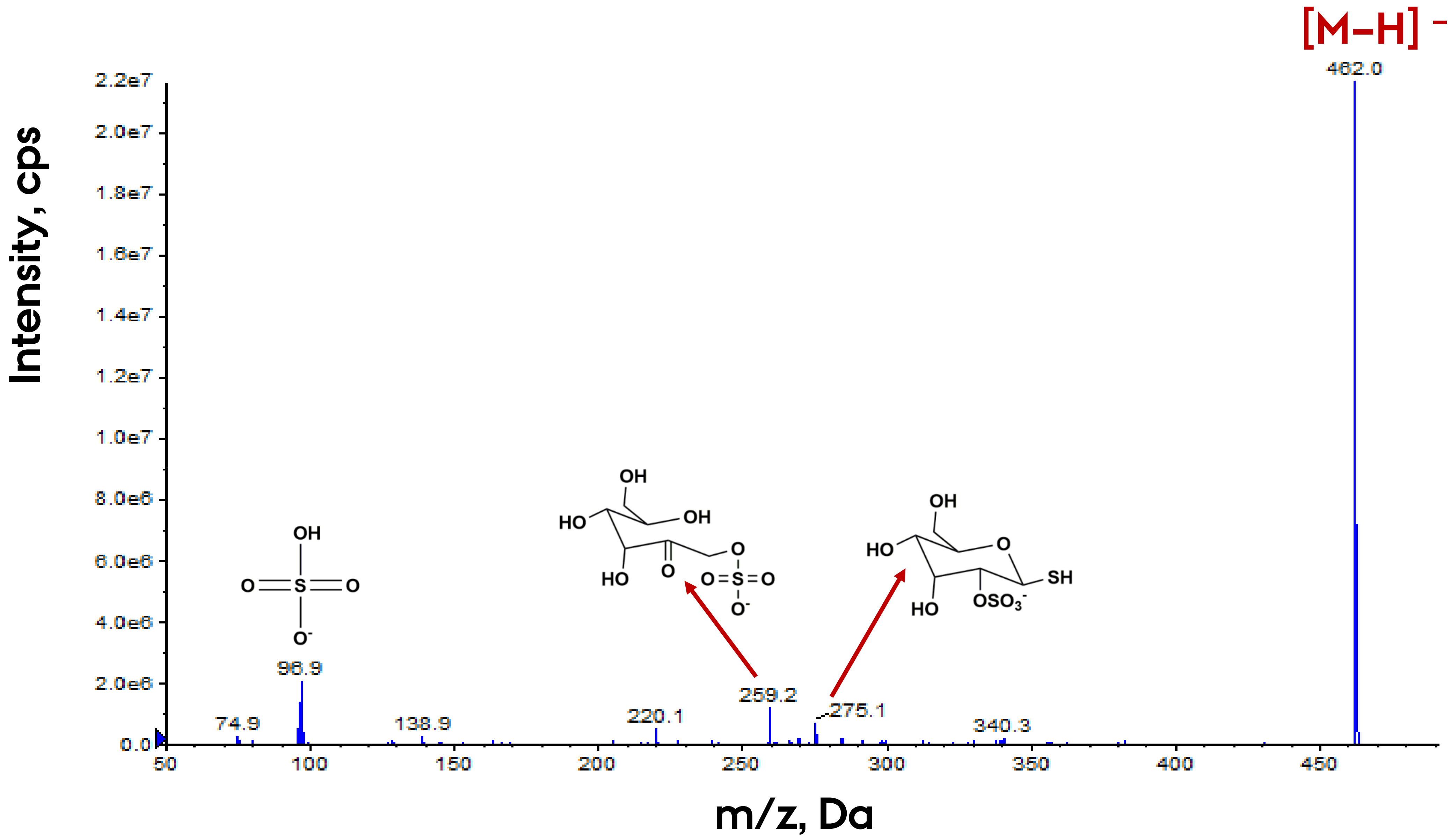

8-methylthiooctyl glucosinolate (8mto)

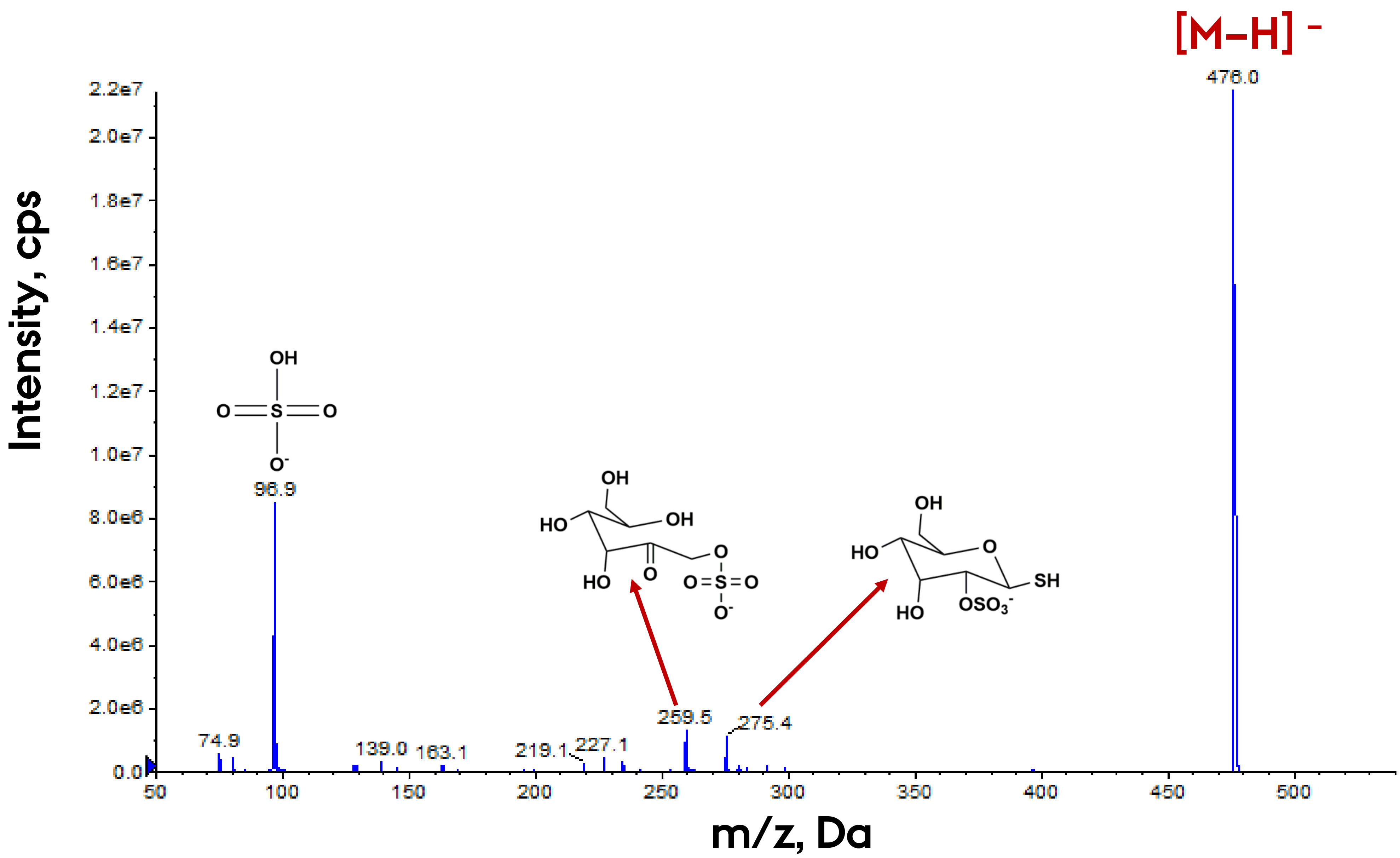

Figure S2. LC–ESI-MS chromatograms of 9 individual glucosinolates (100 ng/ml standard mixture) using a Kinetex 2.6 µm XB-C18 (100x2.1 mm) column. The chromatographic and MS conditions are described in detail in Section 2.1, 3.4, and Table 5.

Visible peaks corresponding to known glucosinolates are numbered according to their elution order on the column: **1**: Glucoiberin (3msp); **2**: glucoraphanin (4msb); **3**: sinalbin (pOHB); **4**: gluconapin (3but); **5**: 4-hydroxyglucobrassicin (4OHI3M); **6**: glucoerucin (4mtb); **7**: glucobrassicin (I3M); **8**: 4-methoxyglucobrassicin (4MOI3M); **9**: neoglucobrassicin (NMOI3M).

Figure S2

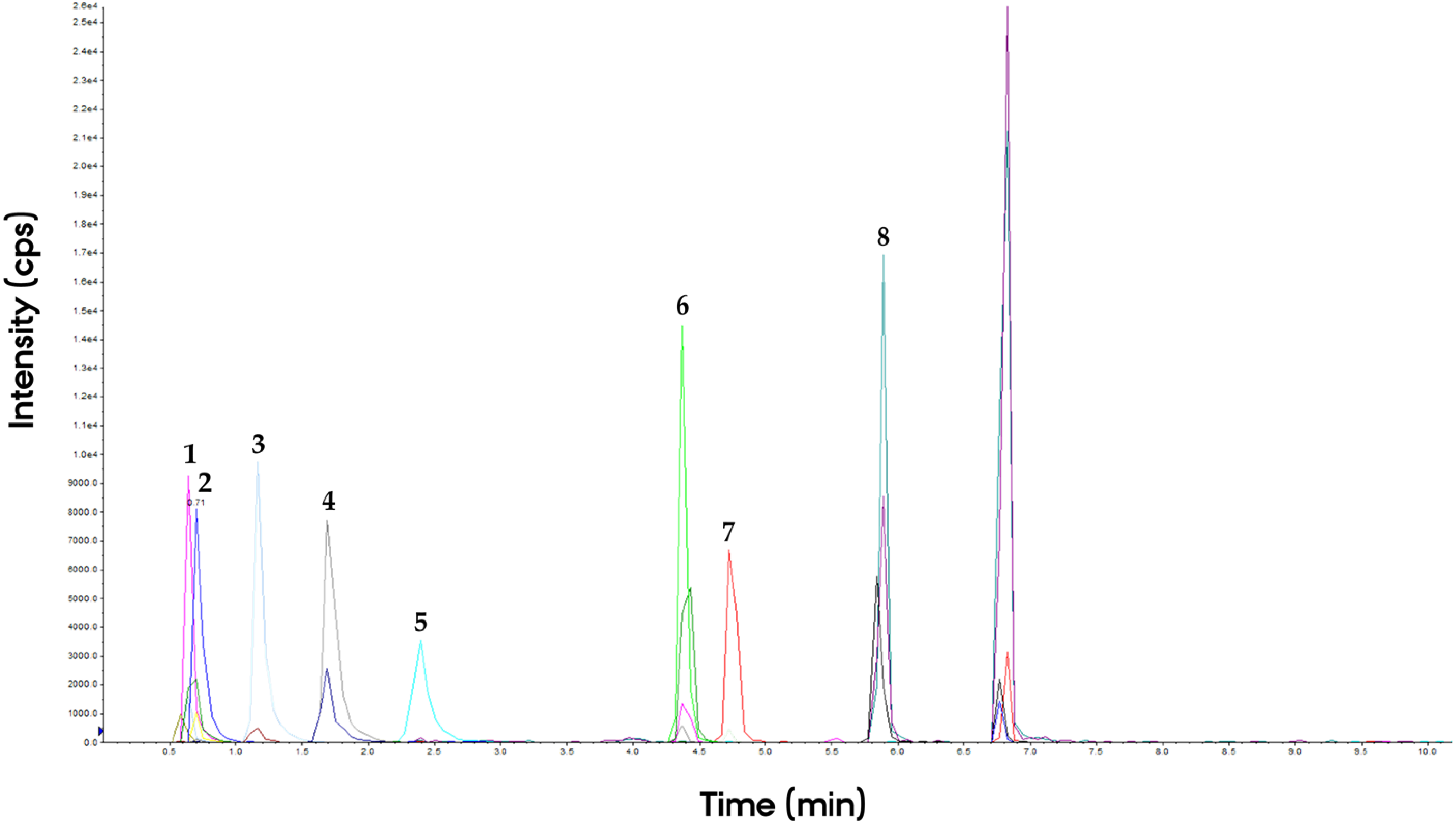

Figure S3. LC–ESI-MS chromatograms of 9 individual glucosinolates (100 ng/ml standard mixture) using a Synergi 4 µm Fusion-RP 80 Å (250 x 2 mm) (Phenomenex) analytical column with a C18 polar embedded functionality. The chromatographic conditions were as follows: **A)** Mobile phase "A" consisted of 100% water with 0.1 acetic acid, and mobile phase "B" consisted of methanol with 0.1% acetic acid. **B)** Mobile phase "A" consisted of 100% water with 0.1% formic acid, and mobile phase "B" consisted of methanol with 0.1% formic acid. **C)** Mobile phase "A" consisted of 100% water with 0.1%acetic acid, and mobile phase "B" consisted of acetonitrile with 0.1% acetic acid. Visible peaks corresponding to known glucosinolates are numbered according to their elution order on the column: **1**: Glucoiberin (3msp); **2**: glucoraphanin (4msb); **3**: sinalbin (pOHB); **4**: gluconapin (3but); **5**: 4-hydroxyglucobrassicin (4OHI3M); **6**: glucoerucin (4mtb); **7**: glucobrassicin (I3M); **8**: 4-methoxyglucobrassicin (4MOI3M); **9**: neoglucobrassicin (NMOI3M).

Figure S3

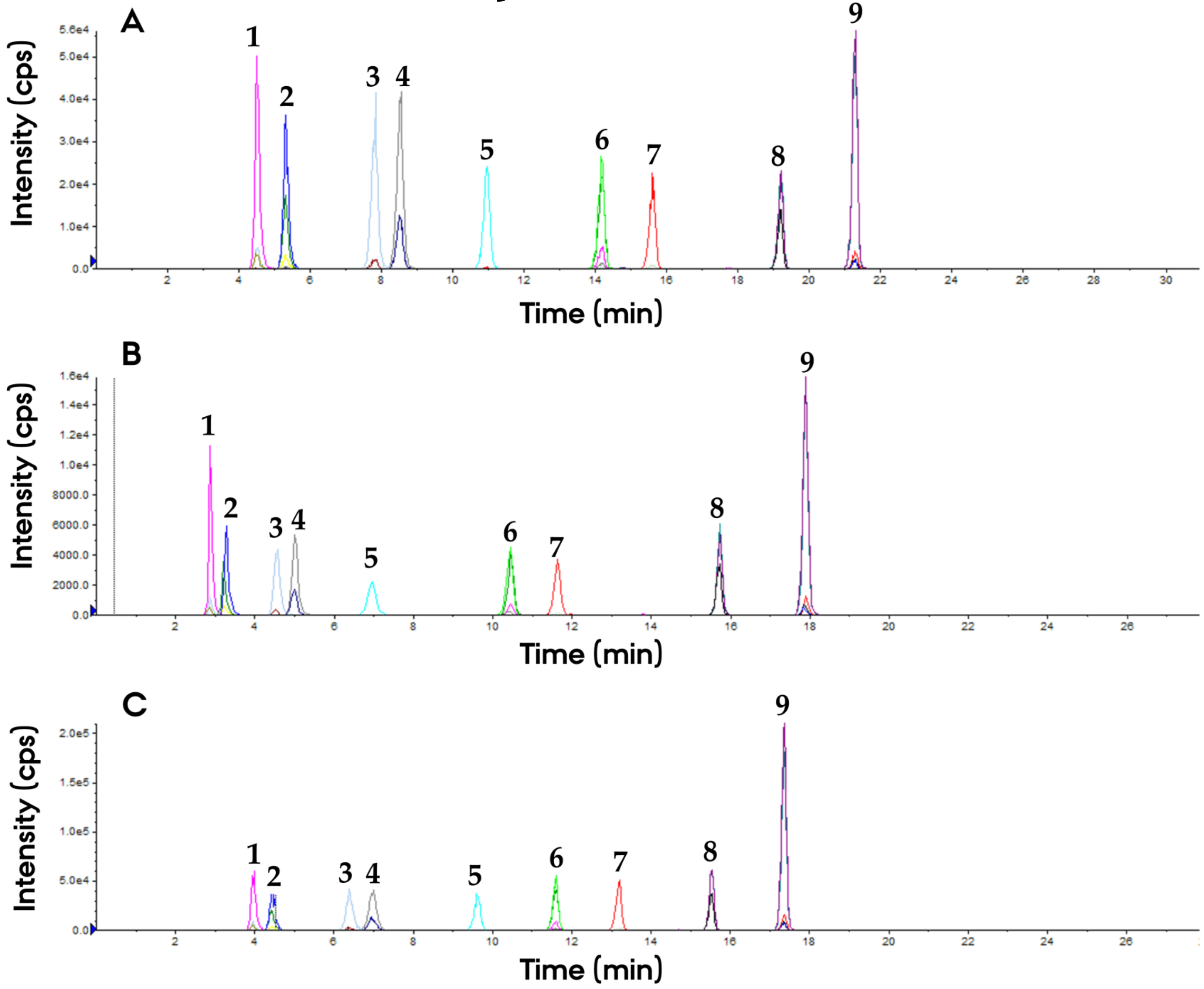

Figure S4. LC–ESI-MS chromatograms of 9 individual glucosinolates (100 ng/ml standard mixture) using three different analytical columns. **A)** Synergi 4 µm Fusion-RP C18 (250 x 2 mm). **B)** Synergi 4 µm Polar-RP 80 Å (250 x 2 mm). **C)** Kinetex 2.6 µm XB-C18 (100 x 2.1 mm). The mobile phase "A" consisted of 100% water with 0.1% acetic acid, and mobile phase "B" consisted of methanol with 0.1% acetic acid. Visible peaks corresponding to known glucosinolates are numbered according to their elution order on the column: **1**: Glucoiberin (3msp); **2**: glucoraphanin (4msb); **3**: sinalbin (pOHB); **4**: gluconapin (3but); **5**: 4-hydroxyglucobrassicin (4OHI3M); **6**: glucoerucin (4mtb); **7**: glucobrassicin (I3M); **8**: 4-methoxyglucobrassicin (4MOI3M); **9**: neoglucobrassicin (NMOI3M).

Figure S4

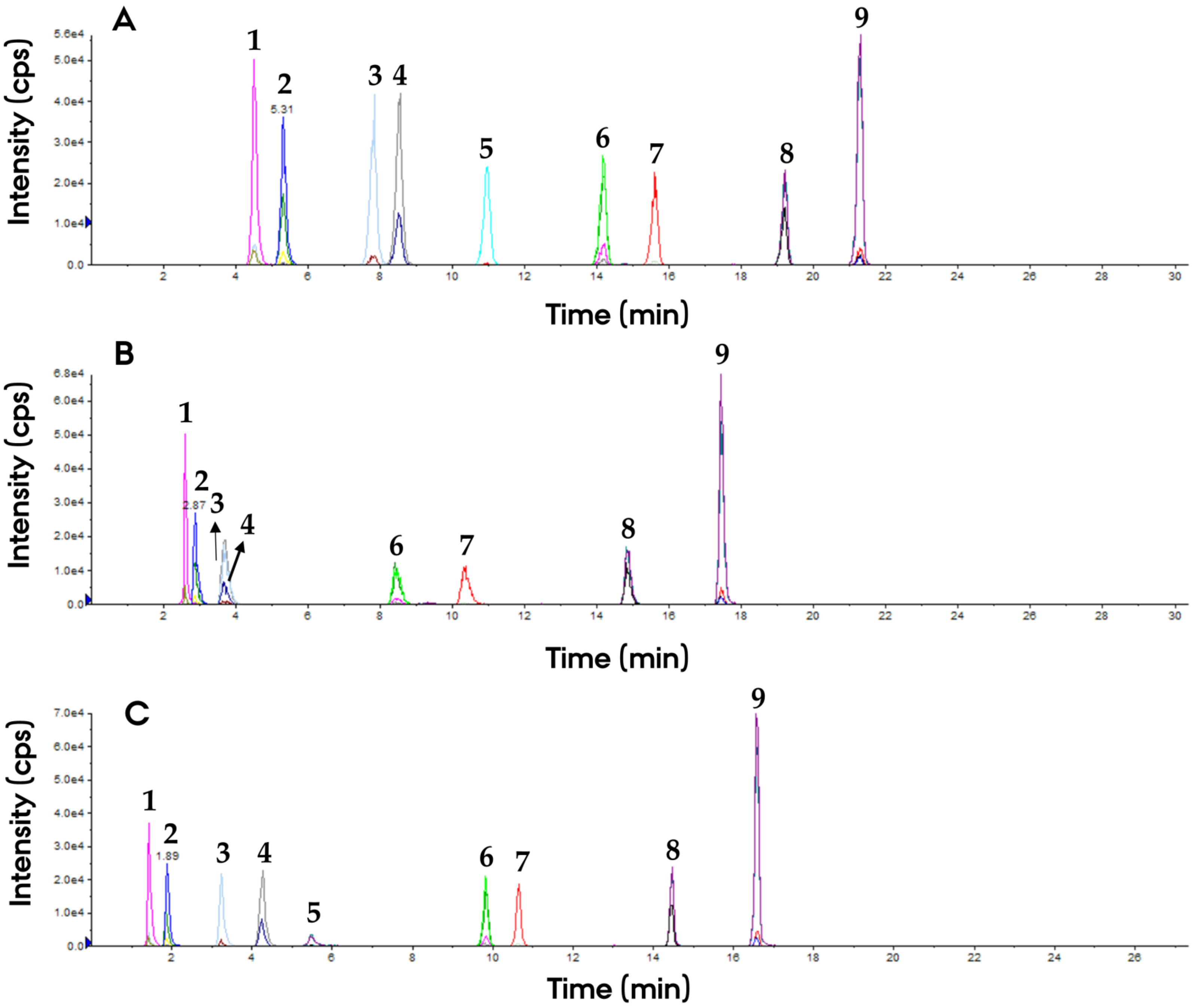

Figure S5. Calibration curves for each of 8 glucosinolates. The 8-point calibration curve (over a concentration range from 1.56 to 200 ng/ml) was constructed by plotting the ratios between the target analyte peak area and internal standard (sinalbin) peak area against the respective concentrations, and the curve was fitted to a linear regression function with a weight of 1/x. The calibration curve was used for the quantitative determination of each target glucosinolate in the samples.

Glucoiberin (3msp)

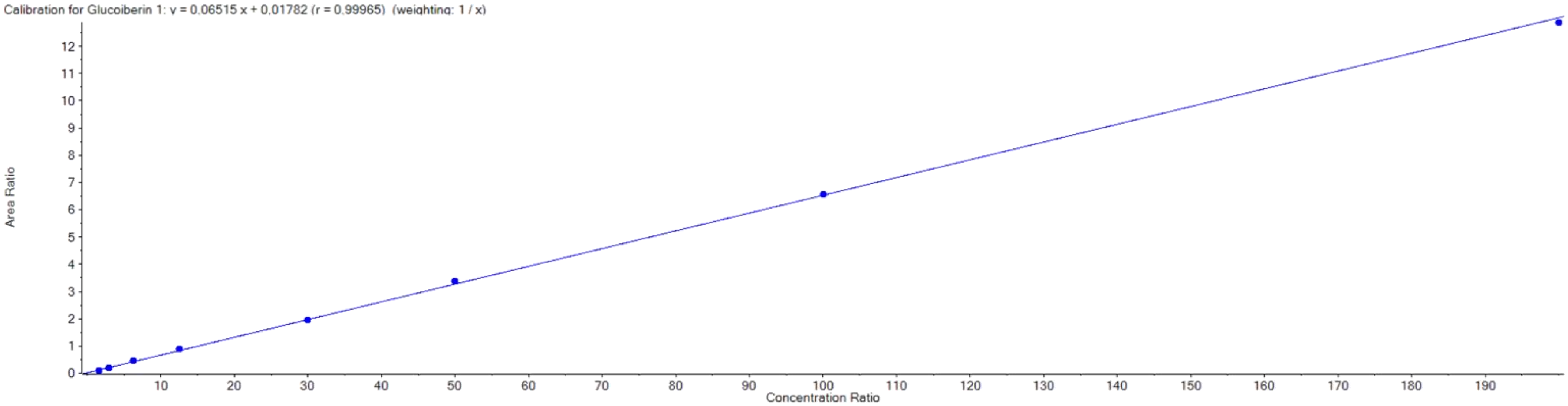

Glucoraphanin (4msb)

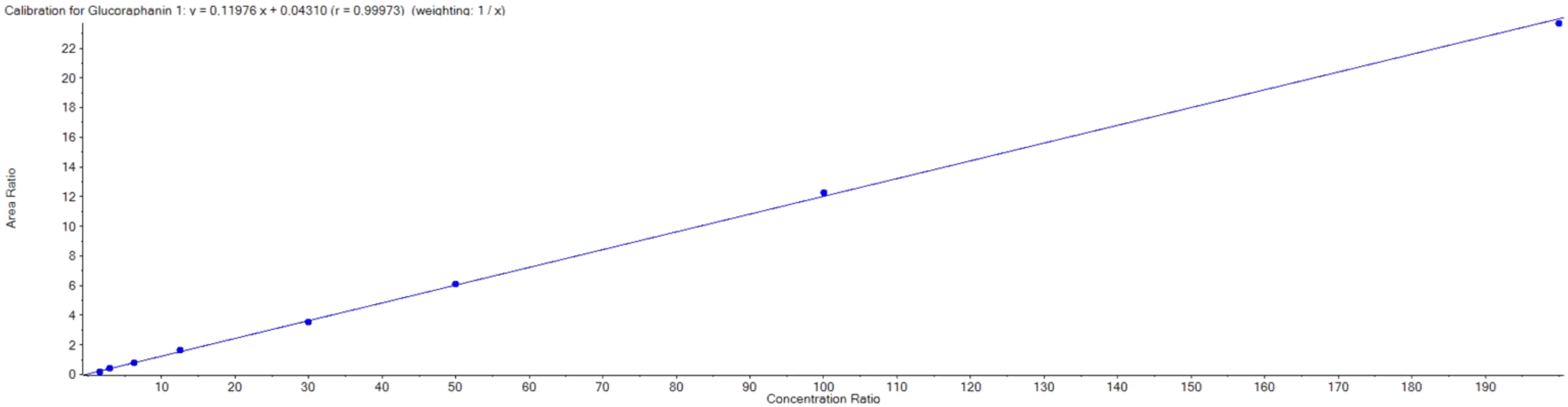

Gluconapin (3but)

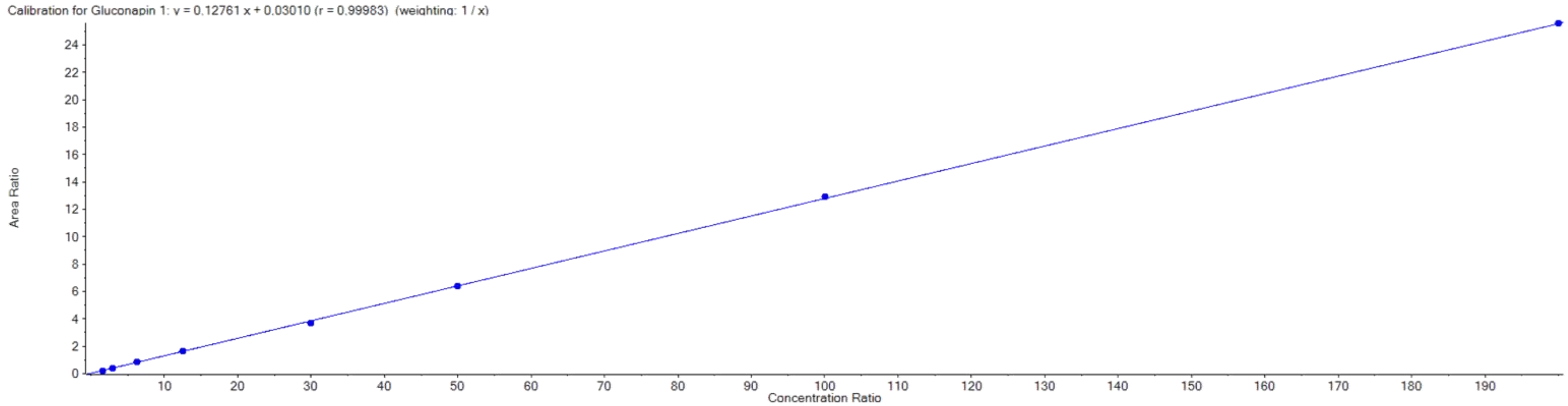

4-hydroxyglucobrassicin (4OHI3M)

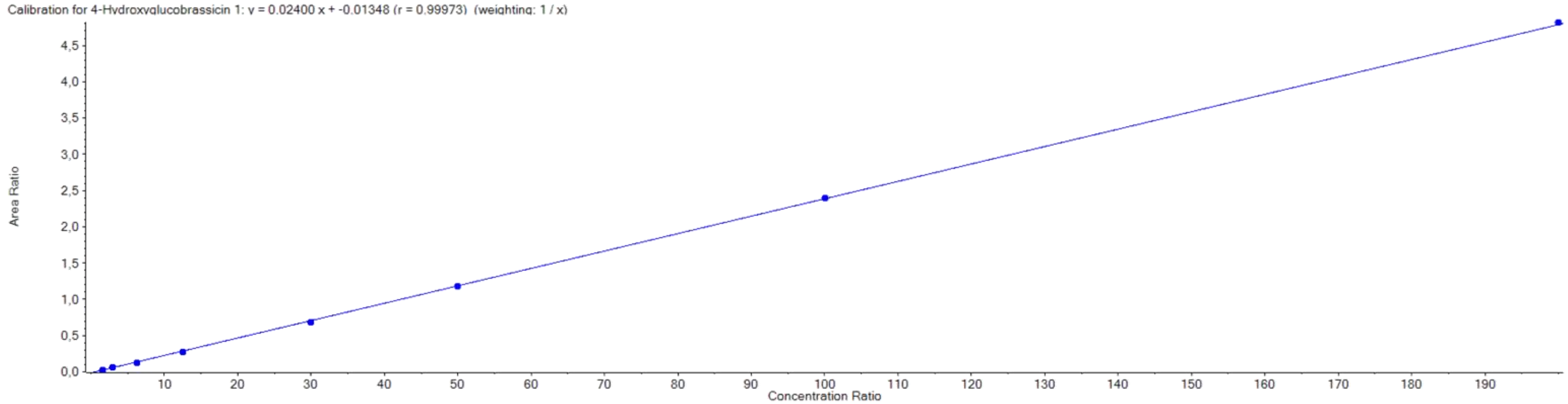

Glucoerucin (4mtb)

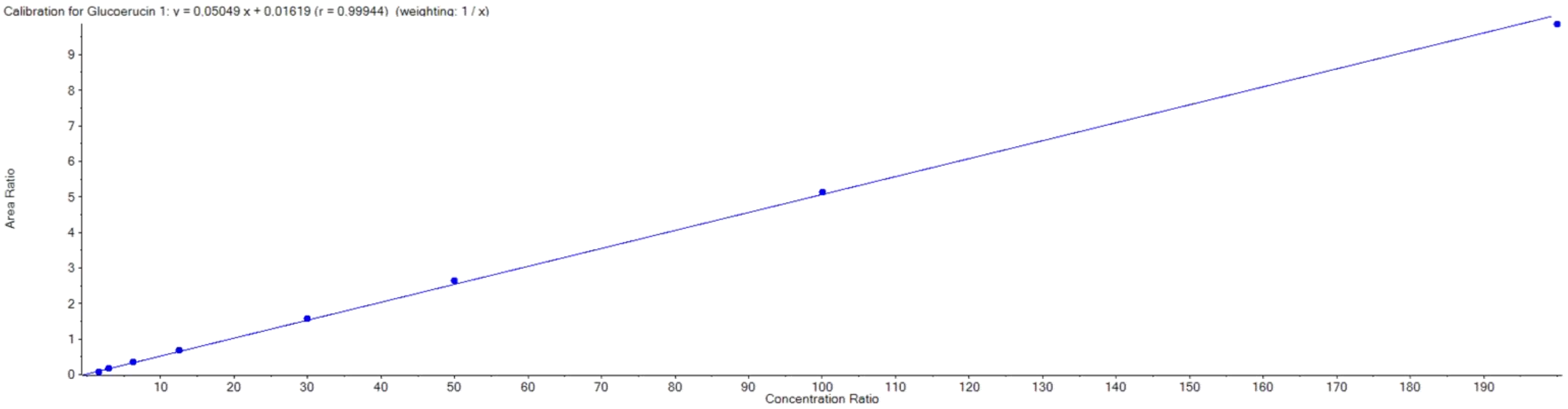

Glucobrassicin (I3M)

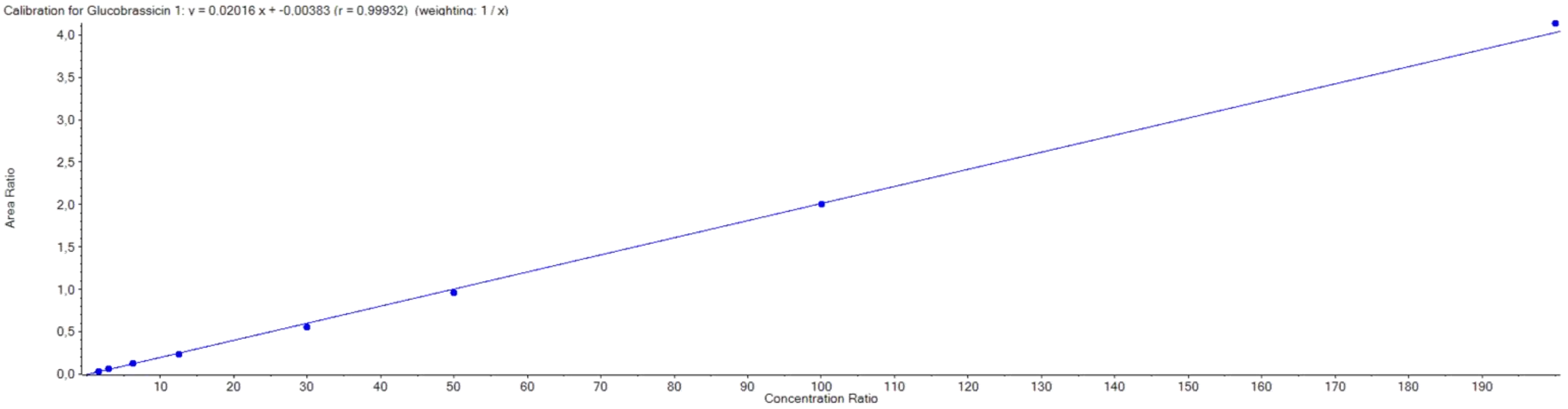

4-methoxyglucobrassicin (4MOI3M)

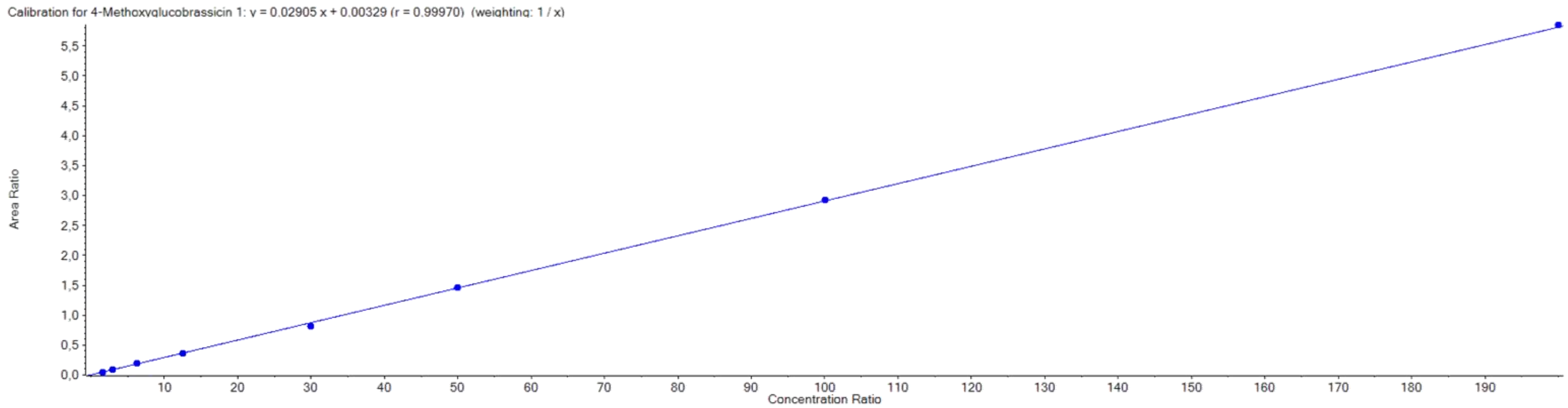

Neoglucobrassicin (NMOI3M)

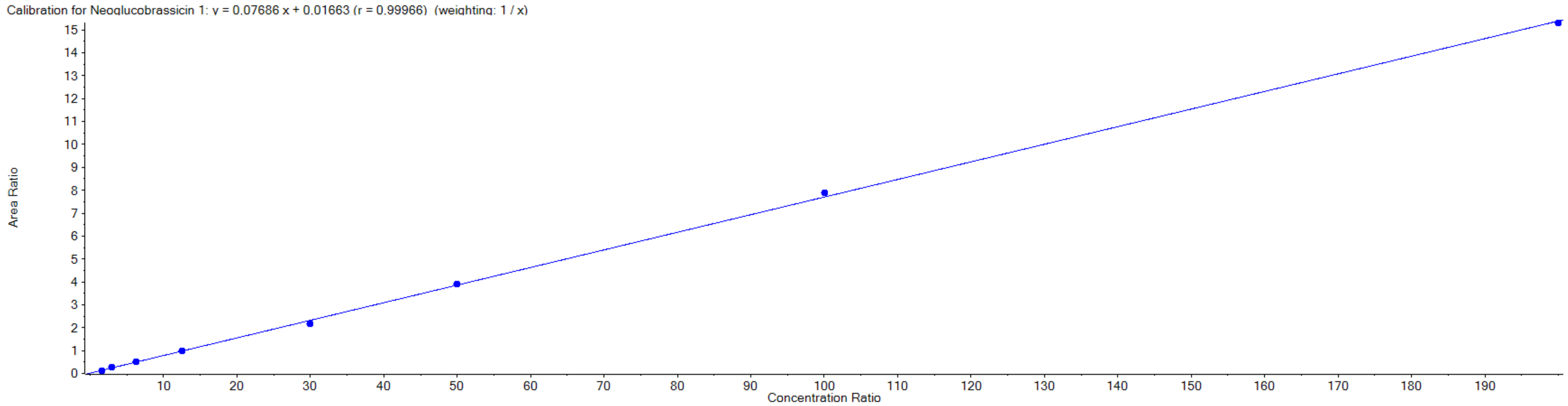

Supplement: Supplementary file 1 [file metabolites-11-00047-s001.pdf]
